# Biochemical Characterization of the *Fusarium graminearum* Candidate ACC-Deaminases and Virulence Testing of Knockout Mutant Strains

**DOI:** 10.3389/fpls.2019.01072

**Published:** 2019-09-10

**Authors:** Thomas Svoboda, Alexandra Parich, Ulrich Güldener, Denise Schöfbeck, Krisztian Twaruschek, Marta Václavíková, Roland Hellinger, Gerlinde Wiesenberger, Rainer Schuhmacher, Gerhard Adam

**Affiliations:** ^1^Department of Applied Genetics and Cell Biology, University of Natural Resources and Life Sciences, Vienna (BOKU), Tulln, Austria; ^2^BOKU, Department for Agrobiotechnology (IFA-Tulln), Institute of Bioanalytics and Agro-Metabolomics, Tulln, Austria; ^3^Department of Bioinformatics, TUM School of Life Sciences Weihenstephan, Technical University of Munich, Freising, Germany

**Keywords:** *Fusarium graminearum*, ACC (1-aminocyclopropane-1-carboxylic acid), ACC synthase, ACC deaminase, ketobutyrate

## Abstract

*Fusarium graminearum* is a plant pathogenic fungus which is able to infect wheat and other economically important cereal crop species. The role of ethylene in the interaction with host plants is unclear and controversial. We have analyzed the inventory of genes with a putative function in ethylene production or degradation of the ethylene precursor 1-aminocyclopropane carboxylic acid (ACC). *F. graminearum*, in contrast to other species, does not contain a candidate gene encoding ethylene-forming enzyme. Three genes with similarity to ACC synthases exist; heterologous expression of these did not reveal enzymatic activity. The *F. graminearum* genome contains in addition two ACC deaminase candidate genes. We have expressed both genes in *E. coli* and characterized the enzymatic properties of the affinity-purified products. One of the proteins had indeed ACC deaminase activity, with kinetic properties similar to ethylene-stress reducing enzymes of plant growth promoting bacteria. The other candidate was inactive with ACC but turned out to be a d-cysteine desulfhydrase. Since it had been reported that ethylene insensitivity in transgenic wheat increased *Fusarium* resistance and reduced the content of the mycotoxin deoxynivalenol (DON) in infected wheat, we generated single and double knockout mutants of both genes in the *F. graminearum* strain PH-1. No statistically significant effect of the gene disruptions on fungal spread or mycotoxin content was detected, indicating that the ability of the fungus to manipulate the production of the gaseous plant hormones ethylene and H_2_S is dispensable for full virulence.

## Introduction

Ethylene is a gaseous plant hormone mediating developmental processes, such as fruit ripening, flower senescence, leaf abscission, as well as root elongation and has a strong influence on growth and yield of crop plants (Dubois et al., 2018). Furthermore, in a complex interplay with other plant hormones, ethylene has an important role in plant immunity (Broekaert et al., 2006; Ent and Pieterse, 2018; Li et al., 2019).

In plants, ethylene is derived from methionine *via* S-adenosyl-L-methionine. In a first dedicated and typically rate limiting step, 1-aminocyclopropane carboxylic acid (ACC) synthase converts S-adenosyl-L-methionine into the precursor ACC, from which ethylene is released by ACC oxidase. Ethylene perception and signalling were elucidated by genetic analysis of the model system *Arabidopsis thaliana*. The hormone is perceived by the copper containing receptor proteins ETR1 and related proteins (Gallie, 2015) that are present in the endoplasmic reticulum membrane. The Raf-kinase like protein CTR1 (Constitutive Triple Response 1) is associated with the ethylene receptor. In the absence of ethylene, CTR1 phosphorylates the ER-localized EIN2 protein at its cytosolic C-terminal part, which is then in the inactive state. When ethylene binds to the receptor, CTR is inhibited and EIN2 becomes dephosphorylated by phosphatases. The C-terminal domain of dephosphorylated EIN2 is then cleaved off and enters the nucleus, which ultimately leads to stabilization of the EIN3 transcription factor against proteasomal degradation (Qiao et al., 2012). EIN3 and the related EIL1 protein subsequently bind to promoters and activate the expression of multiple ethylene-responsive transcription factors (Huang et al., 2016), most importantly of the Ethylene Response Factor 1 (ERF1), which in turn induce the expression of a large number of ethylene responsive genes. With respect to plant defense, genes encoding pathogenesis-related proteins and biosynthetic genes for defense metabolites are induced by ethylene, often in a complex interplay with jasmonic acid. Ethylene and jasmonic acid signaling are considered to mediate defense against necrotrophic pathogens (Glazebrook, 2005) and hemibiotrophic pathogens switching to a necrotrophic mode after an initial biotrophic phase.

Studies with mutants that are deficient in ethylene biosynthesis or are ethylene-insensitive revealed the relevance of ethylene signaling in plant defense, but also the complexity of the interaction (Broekaert et al., 2006). For instance, Arabidopsis *ein2* mutants lost the ability to induce several pathogenesis-related (PR) genes in response to *Alternaria brassicicola* but nevertheless showed unchanged resistance, while the same mutants showed increased susceptibility to *Botrytis cinerea* (Thomma et al., 1999). In transgenic tobacco, which was engineered to become ethylene insensitive by overexpression of a dominant interfering ethylene receptor allele from Arabidopsis (*etr1-1*), breakdown of non-host resistance to several soil fungi was observed (Knoester et al., 1998), and also increased susceptibility to the pathogens *Botrytis cinerea* and *Fusarium oxysporum* (Geraats et al., 2003). Not all aspects of ethylene signaling are conserved between dicotyledonous and monocotyledonous plants (Yang et al., 2015). Nevertheless, ethylene clearly has a role, for instance in defense of rice against the fungal pathogen *Magnaporthe grisea*. Yet, in general, the outcome of virulence tests may be strongly dependent on the combination of host plant and pathogen species (Ent and Pieterse, 2018).

In contrast to the observed trend indicating a role for ethylene in mediating resistance, there are reports that ethylene signaling may also be utilized by fungal pathogens to increase host susceptibility. In the rice interaction with *Cochliobolus miyabeanus* (causing brown spot disease) treatment of plants with the ethylene releasing compound ethephon enhanced susceptibility, while plants with a silenced *OsEIN2* homolog (showing decreased expression of ethylene responsive defense genes) were more resistant (De Vleesschauwer et al., 2010). Similarly, in the interaction between *Fusarium oxysporum* f. sp. *raphani* and *Arabidopsis thaliana*, the ethylene insensitive* etr1-1* mutant was more resistant (Pantelides et al., 2013).

In line with this, the agriculturally relevant pathogen and mycotoxin producer *Fusarium graminearum*, which is in the focus of our interest, was also reported to be less virulent on transgenic wheat with silenced *EIN2* homologs, and importantly, much lower amounts of the mycotoxin DON accumulated in the infected plants. Thus, the fungus may exploit ethylene signaling to promote virulence (Chen et al., 2009). Yet, this is highly controversial. Most gene expression results comparing wheat cultivars of either high or low *Fusarium *resistance found correlations with gene expression consistent with a stronger resistance-associated ethylene-response. A stronger and more rapid response of genes involved in ethylene biosynthesis, ethylene signaling and ethylene response (Ding et al., 2011; Gottwald et al., 2012) and also higher amounts of ACC early in the infection were found in highly resistant cultivars (e.g. Wang et al., 2018). Furthermore, chemical manipulation of ethylene signaling revealed that treatment of highly resistant wheat cultivars with ethylene biosynthesis inhibitors (1-methylcyclopropene and cyclopropane-1,1-dicarboxylic acid) negatively affected resistance to initial infection and disease spread, while treatment with compounds enhancing ethylene production (ethephon and ACC) increased resistance of wheat to *F. graminearum* in highly susceptible cultivars (Foroud et al., 2018).

Several microbes are capable to synthesize ethylene and can consequently trigger ethylene dependent gene expression. In contrast, some microbes can downregulate ethylene production (reviewed in Ravanbakhsh et al., 2018). Fungi can form ethylene at unphysiologically high methionine concentrations in the medium by first transaminating methionine to 4-(methylsulfanyl)-2-oxobutanoate, better known by the synonym 2-keto-methyl-thio-butyrate (KMBA). In a second non-enzymatic reaction, KMBA is then cleaved by hydroxyl radicals (e.g. generated from H_2_O_2_ and Fe^2+^) and ethylene is released [see *MetaCyc* Pathway: ethylene biosynthesis III (microbes)]. This unspecific pathway is seemingly the predominant one in the fungus *Botrytis cinerea* (Chagué et al., 2002). In contrast, many bacteria, particularly plant pathogenic *Pseudomonas* species, can synthesize ethylene from oxoglutarate and arginine *via* the Ethylene Forming Enzyme (EFE). This enzyme is also present in some fungi [*MetaCyc* Pathway: ethylene biosynthesis II (microbes)], for instance in *Penicillium digitatum* (Johansson et al., 2014) which causes fruit rot in citrus. In addition, some fungi, e.g., *Penicillium citrinum* (Kakuta et al., 2001) contain ACC synthase and can synthesize ethylene using the plant pathway [*MetaCyc* Pathway: ethylene biosynthesis I (plants)]. Microbes can also have the ability to downregulate ethylene signalling by interfering with ethylene production. For instance, a bacterial type III effector protein (HopAF1 from *Pseudomonas syringae*) reduces ethylene production by targeting methionine recycling in the Yang cycle (Washington et al., 2016). Also, small molecules can be used as effectors, such as the bacterial rhizobitoxin, which directly inhibits ACC synthase (Yasuta et al., 1999; Sugawara et al., 2006). The ethylene precursor ACC, a signal molecule in plants, is transported *via* the xylem and phloem and causes ethylene production in tissue distant to its site of production (Van de Poel and Van Der Straeten, 2014). The most widely employed mechanism of plant associated bacteria (Gamalero and Glick, 2015; Nascimento et al., 2018) to downregulate ethylene production is the production of ACC deaminase enzymes, which degrade ACC into α-ketobutyric acid (KBA) and ammonia. Reduction of “stress-ethylene” *via* ACC deaminase has been found to be the mode of action of plant growth promoting rhizobacteria that improve the ability of plants to cope with different abiotic stresses, like salt stress (Singh et al., 2015; Qin et al., 2016) or heavy metal stress (Grichko et al., 2000). Expression of ACC deaminase in transgenic tomato was sufficient to enhance growth despite increased heavy metal accumulation (Grichko et al., 2000). ACC deaminases were found and characterized also in several plant associated fungi, for instance in the pathogen *Penicillium citrinum* (Jia et al., 2000), and the biocontrol strain *Trichoderma asperellum* (Viterbo et al., 2010). Such genes have been detected in many fungal genomes, and phylogenetic analysis has provided evidence for multiple independent acquisitions of bacterial ACC genes by horizontal gene transfer (Bruto et al., 2014).

Our research group is interested in virulence mechanisms of *Fusarium graminearum*. Due to the reported ability of this fungus to exploit ethylene signaling for virulence (Chen et al., 2009) and the controversial proposed roles of ethylene in Fusarium head blight resistance, we started to search for genes in *Fusarium graminearum* potentially involved in either ethylene production or downregulation of ethylene signaling, and to experimentally test candidate genes by heterologous expression and gene disruption.

## Materials and Methods

### Bioinformatical Analysis

For the bioinformatic analysis of the genome annotation of *F. graminearum* (PH-1) the PEDANT genome database was used (Walter et al., 2009). The FGSG gene models are still available at http://gbrowse.boku.ac.at/cgi-bin/gb2/gbrowse/Fusarium_graminearum_PH1/and in FungiDB (https://fungidb.org/fungidb/). BLAST searches were performed at NCBI (https://blast.ncbi.nlm.nih.gov/Blast.cgi).

#### Cloning of *Fusarium graminearum* ACS and ACD candidate genes in *E. coli* DH10B

Both *ACD* candidate genes were amplified from gDNA of *F. graminearum* PH-1 using fusion PCR. All PCRs were done as follows: 2 min/95°C initial denaturation followed by 25 cycles of 30 s/95°C, 30 s at primer-dependent calculated annealing temperatures, 1 min/kb at 72°C and a single 72°C/5 min step at the end. All primers and fragment lengths are listed in **Supplementary Table 1**. The two *ACD* candidate genes were cloned in pETDuet-1. The coding region of *ACD1* (FGSG_02678) was cloned *via* BamHI/NotI, and *ACD2* (FGSG_12669) as EcoRI/NotI fragment. Plasmid pET30-UW4-651, containing an ACC deaminase from *Pseudomonas putida* was kindly provided by Prof. Glick, Waterloo, Ontario (Hontzeas et al., 2004). The BglII/HindIII fragment from this plasmid containing the ACD coding sequence was cloned into pETDuet-1 and into pACYCDuet-1 (BamHI/HindIII), a plasmid containing P15A ori (compatible with pETDuet-1) and a chloramphenicol resistance marker.

*ACS1* (FGSG_05184) was amplified from genomic DNA due to the lack of introns, while *ACS2* (FGSG_07606) was amplified from cDNA which was prepared from mycelium harvested from PDA medium according to the protocol of the “RevertAid H Minus First Strand cDNA Synthesis Kit” (Thermo Scientific, Vienna, Austria). *ACS3* (FGSG_13587) was cloned from genomic DNA by fusion PCR. First the exons were amplified with the respective primers shown in **Supplementary Table 1**. Due to the overhangs which are homologous with the primer for amplification of the adjacent exon, amplification of two fused exons was achieved by fusion PCR. *ACS1* was cloned in pET-Duet1 into MCS1* via *EcoRI/NotI while ACS2 and ACS3 were cloned using BamHI/NotI. The plasmids are listed in **Supplementary Table 4**.

#### Expression of *ACS* Candidate Genes in *E. coli*

After induction of transformed *E. coli* strain BL21/DE3 with 1 mM IPTG cells were harvested by centrifugation and the pellets were resuspended in 0.1 M Na-phosphate buffer pH 7.6. For cell disruption a Branson Sonifier W-250 D (Branson Ultrasonics Corporation, Danbury, CT, USA) was used with the following settings: 12 x 5 s pulse and 1 min pause. After centrifugation at 18,000 g the proteins were purified from the supernatants by affinity chromatography using a HisTrap^©^ FF crude 1 ml column using an ÄKTA purifier (GE Healthcare, Austria). For rapid preparations His-select^®^ spin columns (H7787, Sigma-Aldrich, Vienna, Austria) were used.

#### Tests of ACC Synthase Activity

The *ACS* candidate genes were expressed in *E. coli* and ACC synthase activity was tested in living cells by measuring the conversion of methionine into ACC. For this IPTG-induced cultures were supplemented with methionine to a final concentration of 100 mg/l. Samples were drawn after 0, 0.5, 1, 3 and 24 h. The cells were harvested by centrifugation, resuspended in 0.1 M HEPES-KOH buffer pH 8.5 and disrupted by sonication as described above. The concentrations of methionine and ACC in the culture supernatant and in the soluble cell extract were measured by GC-MS.

For *in vitro* tests the proteins were purified using a rapid procedure (His-Select spin columns) according to the manufacturer’s instructions. The activity of the ACC synthases was tested in presence of 50 mM HEPES-KOH buffer (pH 8.5) with 200 µM S-adenosyl-L-methionine (A7007, Sigma-Aldrich, Vienna, Austria) and 10 µM pyridoxal phosphate (P9255, Sigma-Aldrich, Vienna, Austria). Samples were taken every 30 min for 3 h and then after 16, 20, and 24 h and measured by GC-MS.

### GC-MS Measurements of ACC and KBA

The standards for 1-aminocyclopropane-carboxylic acid (ACC; EMD 149101-1G) and 2-ketobutyric acid (KBA; K401, Sigma-Aldrich, Vienna, Austria) were purchased from Sigma-Aldrich. The solvents methanol LC-MS grade (Honeywell 34966) and pyridine p.A. (Merck 1.09728.0500) were obtained from Merck. The derivatisation chemicals methoxyamine hydrochloride (MOX; 226904, Sigma-Aldrich, Vienna, Austria) and N-methyl-N-trimethylsilyl trifluoroacetamide (MSTFA; 701270.201, Macherey-Nagel) were purchased from Sigma Aldrich and Macherey-Nagel, respectively. The stock solutions of ACC and KBA were prepared in 50% aqueous methanol.

The unpurified protein extracts were centrifuged for 10 min at 14,000 rpm at 4°C, 10 μL of the supernatant were transferred into micro-inserts in GC/HPLC vials and dried overnight using a centrifugal evaporator (Labconco, Kansas City, MO) at 15°C. Subsequently an automated two step derivatisation was carried out using the GC auto sampler (PAL LHX-xt, CTC Analytics, Carrboro, NC). To this end the residue was resuspended in 50 µL MOX (20 mg/ml pyridine) and agitated for 90 min at 90°C. After addition of 50 µL MSTFA the mixture was again agitated at 90°C for 60 min.

For GC-MS analysis, 1 µL of the derivate was injected into the split-/splitless injector of an Agilent 7890A gas chromatograph coupled to a 5975C inert XL MSD detector (Agilent, Waldbronn, Germany), equipped with ChemStation software (version E.02.01.1177) and operated in pulsed splitless mode at 250°C. Chromatographic separation was carried out on an HP5-ms column (30 m x 0.25 mm x 0.25 µm; Agilent Technologies, Waldbronn, Germany) at a constant flow of 1 ml/min helium. Temperature program: 50°C, 2 min hold, to 120°C (10°C/min, 5 min hold), to 150°C (5°C/min), to 325°C (70°C/min, 10 min hold). The MSD was operated with interface set to 335°C, the EI source to 230°C and the MSD quadrupole to 150°C, solvent delay 6 min, scan range of *m/z* 50–300, dwell time 100 ms. SIM mode was used for quantification of the ACC 1TMS derivate *m/z* 83 (quant ion), 130, 173; ACC 2TMS derivate *m/z* 147 (quant ion), 202, 230 and KBA 1TMS derivate *m/z* 89, 172, 188 (quant ion).

### Ethylene Measurements

Ethylene (Ethylen Ecocyl 2.5) was purchased from Linde. Ethylene concentrations were determined by GC-FID (Hewlett Packard, Series 2, 5890 Series 2 plus, Agilent, Santa Clara, USA) coupled to a head space autosampler (Hewlett Packard, HP 7694, Agilent, Santa Clara, USA) using the following parameters. Headspace sampler: Oven, sample loop and transferline temperature 45°C, loop 3 ml, loop fill time 0.1 min and injection time 0.2 min; vial parameters: equilibration time 0.5 min, pressurizing time 0.2 min, GC cycle time 11 min. Chromatographic separation was carried out on a Restek Rt-QS-Bond column (30 m × 0.53 mm × 20 µm), using Helium as carrier gas (3.3 ml/min constant flow). Temperature program: 30°C, 1 min hold, to 60°C with 30°C/min, 3.5 min hold. Split injection (30:1) was used and the injector and the flame ionization detector (FID) were set to 250°C. Quantification was based on peak heights and external calibration (10 concentration levels between 0.17 µg/L and 10.65 µg/L). Calibration results were used to estimate the limit of detection (LOD = 0.67 µg/L) and limit of quantification (LOQ = 2.3 µg/L) with the ValiData software Version 3.02.48. Data were processed using MassHunter (Agilent Technologies, Qualitative Analysis B.06.00). Peak areas of the quantification ions were used for comparative quantification and the compound identity was confirmed by comparison with MS spectra of reference standards.

### *In Vitro* Test of ACC Deaminase Candidate Genes

To follow the fate of ACC and KBA in liquid cultures of the *E. coli* BL21/DE3 strain transformed with empty vector or ACD expression vectors, a feeding experiment was performed. The empty vector control allows tracing of possible metabolization products of the target substances by *E. coli*. Expression of the target genes was induced by adding 1 mM IPTG and incubation overnight at 20°C, 140 rpm. ACC and KBA were added (0.5 mg/ml final concentration) to the IPTG-induced cultures. Samples were taken after 0, 1, 3 and 24 h and analyzed for ACC and KBA by GC-MS.

For characterization of the kinetic properties of the ACC deaminase from *Fusarium graminearum* a colorimetric assay relying on the reaction of phenylhydrazine with a ketone to phenylhydrazone was used (Penrose and Glick, 2003). The assay contained purified ACD2 (0.5 mg/ml), ACC concentrations ranging from 0.1 to 100 mM as well as 100 mM Na-phosphate buffer pH 7.6. A calibration curve was generated using KBA.

### d-Cysteine Desulfhydrase Assay of ACD1 With d-Cysteine and l-Cysteine

To test ACD1 (later renamed DCS1 since the protein has d-cysteine desulfhydrase instead of ACC deaminase activity) for activity, 5 µl of the raw protein extract, which was obtained by sonification of IPTG induced cells, were tested in a total volume of 60 µl containing 0.1 M Tris–HCl pH 7.6 and 8 mM d-cysteine and incubation at 37°C for 15 min. The colorimetric assay was performed according to Penrose and Glick (2003), using pyruvic acid for calibration. ACD1 was purified by liquid chromatography *via* the His-tag using HisTrap^™^ columns (GE29-0510-21, Sigma Aldrich, Vienna, Austria). Affinity purified ACD1 was tested for substrate specificity in 50 mM phosphate buffer pH 7.6 using 8 mM of the respective substrate, d-cysteine, l-cysteine, 2-aminoethyl-l-cysteine, or water as negative control. For determination of kinetic properties 0.1–30 mM d-cysteine as substrate were used. All reactions were incubated for 5 min at 30°C and stopped by the addition of 900 µl 0.56 M HCl.

### *ilvA* Knock-Out in *E. coli* T7-Express

In order to be compatible with the T7 polymerase based expression system we knocked out *ilvA* in the expression host strain. The resulting strain is auxotrophic for isoleucine due to the loss of the ability to synthesize KBA, an intermediate of isoleucine biosynthesis. We obtained the *ilvA* mutant *E. coli* strain JW3745-2 from “The Coli Genetic Stock Center.” The kanamycin resistance gene replacing the *ilvA* gene was amplified with primers 5´CGGAGATGTGGTAGTAATTC-3´ and 5´-GCCGTTTATTATGGCCGATC-3´, which bind in the neighbouring genes *ilvD* and *ilvY*. The 2085 bp fragment was purified and adjusted to a final concentration of 100 ng/µl. The strain to be transformed, *E. coli* T7-express (NEB, Frankfurt am Main, Germany), was first endowed with plasmid pKD46, which contains an arabinose inducible phage lambda *red* recombinase (Datsenko and Wanner, 2000). The PCR product (100 ng) was used for electroporation followed by selection on LB+kanamycin plates at 37°C, leading also to the loss of the temperature sensitive pKD46. Kanamycin resistant mutants unable to grow on M9 plates were tested by PCR. The resulting strain—T7 express *ilvA*::*Kan* *^R^*—is available upon request. This strain was further transformed with the empty pET-DUET-1 vector or with pET-DUET-1 containing either ACD1, ACD2, or the positive control of *Pseudomonas putida* in MCSI. Expression was induced by IPTG (1 mM final concentration).

The positive control from *P. putida* (*ACDP*) was released from pET30a by BglII/HindIII cleavage and cloned into BamHI/HindIII digested pACYCDUET-1. This vector was transformed together with one of the *ACS* genes in pETDUET-1 into the generated *E. coli* T7-express lacking *ilvA*. *ACS4* of *Arabidopsis thaliana* (AT2G22810.1) in pETDUET-1 served as a positive control. Transformants were selected on LB plates containing ampicillin (100 mg/l) and chloramphenicol (25 mg/l). For the spottings, M9 was supplemented either with 3 mM methionine or 3 mM ACC.

### Preparation of Knock-Out Mutants in *F. graminearum*

PEG-mediated protoplast transformation was performed as described by Twaruschek et al. (2018). For gene disruptions the split marker strategy was applied and fragments for transformation were produced as described below. The 3´ and 5´ flanking region of the candidate genes were cloned in pASB42 (see Twaruschek et al., 2018) adjacent to *hph*-*amdS* cassette flanked by two loxP sites (Steiger et al., 2011) using the primers listed in **Supplementary Table 2**. Screening of transformants was performed as described in (Twaruschek et al., 2018) using the primers listed in **Supplementary Table 3**. For multiplex PCR, three primers which are either located outside of the flanking region which was used for homologous recombination, or in the resistance cassette, or in the target gene to be disrupted were used. The corresponding primers in the resistance cassette were 5´AGAAGTACTCGCCGATAGTG-3´ for the 5´-flanking region, and 5´-ACACCTGCCGTGTCAGCC-3´ for the 3´-flanking region. The primers were designed in a way that the band of the wild type and the one of the knock-out strain can be easily differentiated.

For generating *acd1 acd2* double mutants, a knock-out construct with HSVtk-nptII as selection marker was generated: *ACS* flanking region containing plasmids pTS24, pTS35 and pTS45 were cut with HindIII/SalI. pTS14 and pTS58 were cut with BcuI/SfiI and pTS61 was digested BglII/BcuI (**Supplementary Table 4**). The HSVtk-nptII cassette from pKT245 was digested the same way to replace amdS-hyg (Twaruschek et al., 2018). Two independent, genotypically identical mutants (strains TS_ACD1Δ_2 and TS_ACD1Δ_13, listed in **Supplementary Table 5**) lacking *ACD1* were transformed using the split marker strategy. For screening of the double knock-out candidates, the same outer primer and the one within the gene as for the single knock-out screening were used. Due to a different resistance cassette the primers in the cassette were adapted to 5´-GTAGACCGCAAATGAGCAAC-3´ for the screening of the 5´ region, and 5´-GCCACAGCAGCCACGACA-3´ for the 3´ region. The resulting strains have the genotype *dcs1*Δ::*loxP-hyg-amdS-loxP* *acd2*Δ::*loxP-nptII-HSVtk-loxP* (**Supplementary Table 6**). Six PCR confirmed double-knockout strains were chosen for the virulence test with 10 replicates per strain. The progress of infection was observed over a time period of 16 days followed by analysis of DON and D3G after harvesting.

### Test of Utilization of ACC as Sole Nitrogen Source

Fusarium Minimal Medium (FMM, for recipe see Twaruschek et al., 2018) was modified by replacing NaNO_3_ with 3 mM ACC. Suspensions containing 10^5^ spores were spotted onto the plates, which were incubated for 10 days at 20°C in the dark.

### Wheat Infections

For the infection assays the cultivar Apogee (Mackintosh et al., 2006) was used. Ten microliters of a spore suspension with 4*10^4^ spores/ml was pipetted into each of four florets of two spikelets in the middle of the ear. Moistened plastic bags were used to cover the infected ears for 24 h to maintain high humidity. Plants were kept in a growth chamber (20°C with 16 h/8 h light/dark periods) and the disease progression was observed over a time period of 16 days by counting the infected spikelets every other day. For toxin analysis the ears at the endpoint of 16 days were harvested, frozen in liquid nitrogen and ground in a Retsch mill (Retsch MM400) using steel balls. For extraction 400 µl solvent (acetonitrile/water/acetic acid = 79: 20: 1) per 100 mg sample was added. The samples were extracted for 1 h at 20°C, 180 rpm, and centrifuged for 10 min at 14,000 g, and a 1:10 dilution was prepared for analysis by HPLC-MS.

#### Quantitative Analysis of DON and D3G

Deoxynivalenol (DON) and DON-3-O-glucoside (D3G) were determined by HPLC-MS/MS. A 1290 UHPLC system from Agilent Technologies (Waldbronn, Germany) equipped with Gemini C18 column (150 × 4.6 mm, 5 µm; Phenomenex, Aschaffenburg, Germany) was used for separation of analytes. The mobile phase eluents were composed of water and methanol (A: 80:20, v/v; B: 3:97, v/v) and contained both 5 mM ammonium acetate. The applied gradient was as follows: 0–1 min (0% B); 1–6 min (linear gradient from 0% B to 50% B); 6.1–8 min (flushing of the column with 100% B), 8–10 min (column equilibration with 0% B). The chromatographic system was maintained at 25°C and the flow rate of mobile phase was set to 800 µl/min.

Detection and quantification of both target analytes were performed on mass spectrometer QTrap 4000 (Sciex, Foster City, CA, USA), equipped with a TurboV electrospray ionization source. The system was operating in negative electrospray ionization mode (ESI-). The following source parameters were applied: curtain gas 35 psi (240 kPa), ion spray voltage (4 kV), temperature 550°C, ion source gas 1 and 2 both 50 psi (344 kPa), collision gas (nitrogen) high, and the interface heater on. The following selected reaction monitoring (SRM) transitions with a dwell time of 25 ms were used: DON (retention time 5.75 min) m/z 355.1 (declustering potential, DP, 65 V), product ions m/z 265.2 (collision energy, CE, 28 V) and m/z 59.2 (CE, 21 V); D3G (retention time 5.45 min) m/z 517.3 (DP, 56 V), product ions m/z 427.1 (CE, 25 V) and m/z 59.1 (CE, 18 V). Data were processed with Analyst 1.6.3 software from Sciex and further data processing and calculation of concentrations were performed using Microsoft Excel 2010. The limits of detection and quantification for DON and D3G were 10 and 25 ng/ml, respectively.

### Cysteine Racemase Test

To check whether *F. graminearum* is able to convert l-cysteine into d-cysteine or vice versa, protein extracts were tested for racemase activity. Pre-cultures were prepared by inoculation of 10^5^ spores of strain PH-1 in 100 ml FMM medium. After 3 days of incubation the mycelia were harvested using a sterile filter funnel, washed and inoculated in modified FMM (MgSO_4_ replaced by MgCl_2_ and supplemented with 1 mM d- or l-cysteine). The cultures were incubated at 20°C overnight followed by filtration of the mycelium. The mycelia were blotted dry, frozen in liquid nitrogen, pulverized using Retsch mill and 2 µl 50 mM Na-phosphate buffer pH 6.6 was added per milligram mycelium followed by vortexing 3 × 30 s. The protein extracts were centrifuged and the supernatant was used.

The assay contained 20 µl of the protein extract, 75 µl 50 mM phosphate buffer pH 6.6, and 25 µl d- or l-cysteine (10 mM final). The negative controls were supplemented with buffer instead of cysteine. Samples were taken after 0 and 24 h by transferring 50 µl of the samples into a tube containing 150 µl MeOH. One volume of ice-cold chloroform and four volumes of ice-cold methanol were added. The mixtures were shaken at 4°C for 15 min and precipitated proteins were pelleted by centrifugation at 12,000 g at 4°C for 10 min. The clear aqueous phase was transferred into another 1.5 ml reaction vial. The process was repeated once and the collected methanol phase was evaporated in a centrifugal vacuum evaporator. The dried residue was kept at −20°C until derivatization. Marfey’s reagent (Pierce TS-48895) was used for derivatization of enantiomers to receive diastereomers. This derivatization allows chromatographic separation and detection of isobaric compounds derived from d-/ l-cysteine.

#### Quantification of d-, l-Cysteine Racemization Using LC-HR-MS Measurements

For quantification of d-cysteine desulfhydrase activity and to measure racemase activity an ultra-high performance liquid chromatography (UHPLC) Vanquish system coupled to a QExactive HF Orbitrap (both ThermoFisher) was applied to measure d-/l-cysteine levels. Five microliters of sample extract was injected for chromatographic separation on a Gemini NX-C18 column (150 × 3 mm ID, 5 µm, 110 Å, Phenomenex), which was protected by a pre-column and maintained at 25°C at a constant flow rate of 400 µl/min. To achieve chromatographic separation of the cysteine derivatives a gradient program with linear gradient segments was applied. Buffer A was water containing 0.1% formic acid and buffer B was acetonitrile containing 0.1% formic acid. The gradient program was as follows (% acetonitrile/time in minutes): 10/0, 10/3, 45/56.1, 99/57, 99/62, 10/63, 10/70. UV traces were recorded at 220 and 340 nm.

The MS system was equipped with a heated electrospray ionization (HESI) source. The ESI interface was operated in fast polarity-switching mode, using 55 L/min sheath gas flow, 5 L/min auxiliary gas flow, a spray voltage of 3.5/3.0 kV respectively for the positive and negative ionization mode, S-lens level 55, capillary temperature 320°C and auxiliary gas temperature 350°C. FT-Orbitrap was operated in full scan mode acquiring profile spectra for the scan range m/z 100–1000 with a resolving power of 120,000 FWHM (at m/z 200) and automatic gain control setting of 3x10^6^ with a maximum injection time of 200 ms. The mass analyzer’s mass accuracy was calibrated on a daily basis using Pierce LTQ Velos ESI ion calibration solution (ThermoFisher). A 1:1 mixture of the solution for positive and for negative ionization was prepared for calibration. The system was operated with TUNE 2.8 SP1 software and the direct control plugin for Chromeleon 7.2 SR4. Data processing was performed with XCalibur 4.0.27.19 (ThermoFisher). Quantification of area under the curve for the detected analytes was obtained with the XCalibur plug-in QuanBrowser. The retention times and m/z species used for quantification of the analytes were as follows: Marfey’s reagent 23.80 min, l-cysteine 46.75 min, d-cysteine 49.90 min, D-valine 38.70 min, d8-D-valine 38.60 min, LL-cystine 47.20 min, DD-cystine 53.90 min, and DL-cystine 44.7 min. Note that Marfey’s reagent reacts with both the primary amines and the free sulfhydryl groups in cysteine. The retention time of the diastereomeric amino acid derivatives was determined using pure enantiomeric reference standards (Sigma-Aldrich, Vienna, Austria). Reference materials were prepared in 10 mM HCl and working solution for calibration were diluted in water. An internal standard calibration was applied to account for matrix effects as well as for variation of chemical derivatization with the Marfey’s reagent. Due to the lack of sufficient isotopically labelled D- and L-cysteine and DD-, DL-, and LL-cystine reference standards, the representative amino acid D-valine was chosen as internal standard. Accordingly, quotient of analyte/ISTD area under the curve was calculated for all analytes. The ISTD concentration of 6.94 µM after spiking the analytes into the sample and derivatization was kept constant for all calibration standards and the biological samples. The applied concentrations for cysteine to calculate calibration function and for analyte quantification were 16, 80, 400, 2,000, and 10,000 µM. The obtained calibration function was linear for the chosen concentration range. The limit of detection was estimated in a serial dilution experiment for the analytes with greater than 2.89 µM.

## Results

### Bioinformatical Analysis of the *F. graminearum* Genome—Presence of EFE?

The first question we addressed was whether the genome sequence of *F. graminearum* contains candidate genes that might allow the fungus to synthesize ethylene and thereby exploit ethylene signaling to increase virulence. The EFE pathway is the most efficient mechanism to produce ethylene. Therefore, we started the search for candidate genes using an EFE with biochemically confirmed activity (GenBank: EKV19239.1) as a query in a BLASTP search. As shown by Johansson et al. (2014), the associated mitochondrial import sequence was missed in the genome annotation of *Penicillium digitatum* (Marcet-Houben et al., 2012). The protein sequence in the database starts at the second in-frame ATG and is missing a number of N-terminal amino acids present in the protein sequence of the purified enzymes (see **Supplementary Figure 1**). We therefore used the modified gene model proposed by Johansson et al. (2014), which is highly similar to gene model (XP_002562422.1) from *Penicillium chrysogenum/P. rubens* (van den Berg et al., 2008). The BLASTP search using the N-terminally extended sequence (EKV19239.1) as query against the *F. graminearum* PH-1 genome yielded five hypothetical proteins (FGSG_00893, FGSG_00048, FGSG_03213, FGSG_08081, FGSG_02301) with sequence identities lower than 24% (low E-values below 9e-15, query covers below 75%). These low-similarity genes most likely encode members of the 2OG-Fe(II) oxygenase superfamily (pfam03171) with functions other than ethylene formation. The whole *F. graminearum* species complex (taxid:569360) contained no good candidate *EFE* gene, with the notable exception of *F. venenatum* (XP_025583657.1), having 66.39% identity with a query cover of 99%. The sequences differ to the highest extent in the putative mitochondrial import sequence, which is shorter in *Fusarium*. Interestingly, many other *Fusarium *species, particularly from the *F. oxysporum* and *F. fujikuroi* species complex had also highly similar genes, indicating that *EFE* could be present and conserved in the genus *Fusarium* (taxid:5506) after all (see **Supplementary Figure 1**). Yet, also in other *Penicillium* species, non-functional homologs highly similar to the enzymatically active *P. digitatum* *EFE* exist. Site-specific mutagenesis had revealed that introduction of several amino acids, which are different in the inactive enzyme from *P. chrysogenum, *into the active *Pseudomonas syringae* pv. *phaseolicola* enzyme (P32021.1, Fukuda et al., 1992), leads to loss of activity of the latter (Johansson et al., 2014). The candidate gene from *F. oxysporum* f. sp. *cepae* (RKK90967.1, as published by Armitage et al., 2018), has the highest similarity with the N-terminally extended *P. digitatum* gene (65% sequence identity over 88% of the query sequence). Using this protein sequence as query, the BLASTP results indicate that many gene models of other *Fusarium oxysporum* isolates are probably missing the C-terminal exon and therefore run into a premature stop. Closer analysis of one of these putatively truncated EFE candidates (Accession PCD42226.1) has shown that this is due to a GT → GA mutation in a donor splice site, which is then no longer recognized by automated annotation algorithms. However, studies of *Fusarium *splicing by (Zhao et al., 2013) have shown that the organism does actually use GA as a donor site, therefore the algorithm used to detect these “premature stops” might have overlooked an intron, and the truncated gene models might, in fact, not be truncated at all. **Supplementary Figure 1** shows the respective alignment, indicating that the hypothetical protein AU210_004756 from *Fusarium oxysporum* f. sp. *radicis-cucumerinum* can easily be modified to resemble the non-truncated candidates of other species. A potential exception is the unordered region (blue letters in **Supplementary Figure 1**), which was not included in the structural model of Johansson and coworkers (2014). In summary, we conclude that the *EFE* homologs seem to be widespread in different *Fusarium* species, but functional testing is necessary to find out whether the candidates are indeed encoding active enzymes or not. In contrast, *F. graminearum* and close relatives (*F. culmorum*, *F. pseudograminearum*) do not possess *EFE*.

### Identification of ACC Synthase, Oxidase, and Deaminase Candidate Genes in the *F. graminearum* Genome

Using headspace GC-MS we found increased ethylene production of *F. graminearum* PH-1 on medium with 20 mM methionine added (**Supplementary Figure 2**), which suggests that the ACC synthase pathway might be active. Yet, we cannot rule out that the observed ethylene production is due to the unspecific pathway III found in a broad range of microbes.

We investigated whether *F. graminearum* possesses ACC synthase and ACC oxidase candidate genes. A BLASTP search with the sequence of the enzymatically active enzyme from *Penicillium citrinum* (BAA92149.1, described by Kakuta et al. in 2001) revealed three candidate genes: *FGSG_05184*/*FGRAMPH1_01G17303*, (named *ACS1* in this study), *FGSG_07606/FGRAMPH1_01T25199* (*ACS2*), and *FGSG_13587* (*ACS3*). These genes were already previously annotated as ACC-synthases in the Fusarium Genome Database (FGDB) by Wong et al. (2011). The alignment score between the ACC synthase of *P. citrinum* and *Fusarium* genes is 52.3 for ACS1, 37.1 for ACS2 and 29.8 for ACS3. An alignment of these three predicted proteins with the ACC synthase of *P. citrinum* and an ACC synthase from *Triticum aestivum* (AAB18416.1), which were both confirmed to be active when expressed in *E. coli* (Subramaniam et al., 1996; Kakuta et al., 2001), is shown in **Supplementary Figure 3**. The phylogenetic tree shows that ACS3 is closely related to ACC synthase of *T. aestivum* and ACS1 is closely related to the confirmed gene of *P. citrinum*. The candidate genes have highly conserved residues involved in the catalytic activity and were therefore selected for experimental testing by heterologous expression in *E. coli*.

A BLAST search with a confirmed ACC oxidase from the basidiomycete *Agaricus bisporus* (Swiss-Prot H9ZYN5) showed that *F. graminearum* genome contains two predicted genes, *FGSG_09103* and *FGSG_11522*, with 79% and 88% query cover and 38% and 34% identical amino acids, respectively, while three other hits showed lower similarity. Both of these candidate genes are annotated as “isopenicillin N synthase and related dioxygenases,” and are rather unlikely to have ACO activity, however, confirming this would require experimental testing.

While the results of Chen et al. (2009) suggest that *F. graminearum* exploits signaling for virulence by having the ability to produce ethylene, also, the opposite scenario cannot be excluded. We therefore searched for ACC deaminase candidate genes. A BLASTP search with a biochemically characterized ACC deaminase of *Trichoderma asperellum* (ACX94231.1, Viterbo et al., 2010) showed two hits in which the query cover was 99% and 98%, respectively, with 84% and 42% identical amino acids. These two genes (*FGSG_02678*/*FGRAMPH1_01T06417*, named *ACD1* and *FGSG_12669*/*FGRAMPH1_01T16927*, named *ACD2*) are also annotated as ACC deaminases also in FungiDB. The alignment of the confirmed ACC deaminase of *T. asperellum* with both candidate genes of *F. graminearum* is shown in **Supplementary Figure 4**. The phylogenetic analysis showed that *ACD2* is closer related to the confirmed ACC deaminase of *T. asperellum*.

### Activity Test of *ACS* Candidate Genes

**Figure 1:**
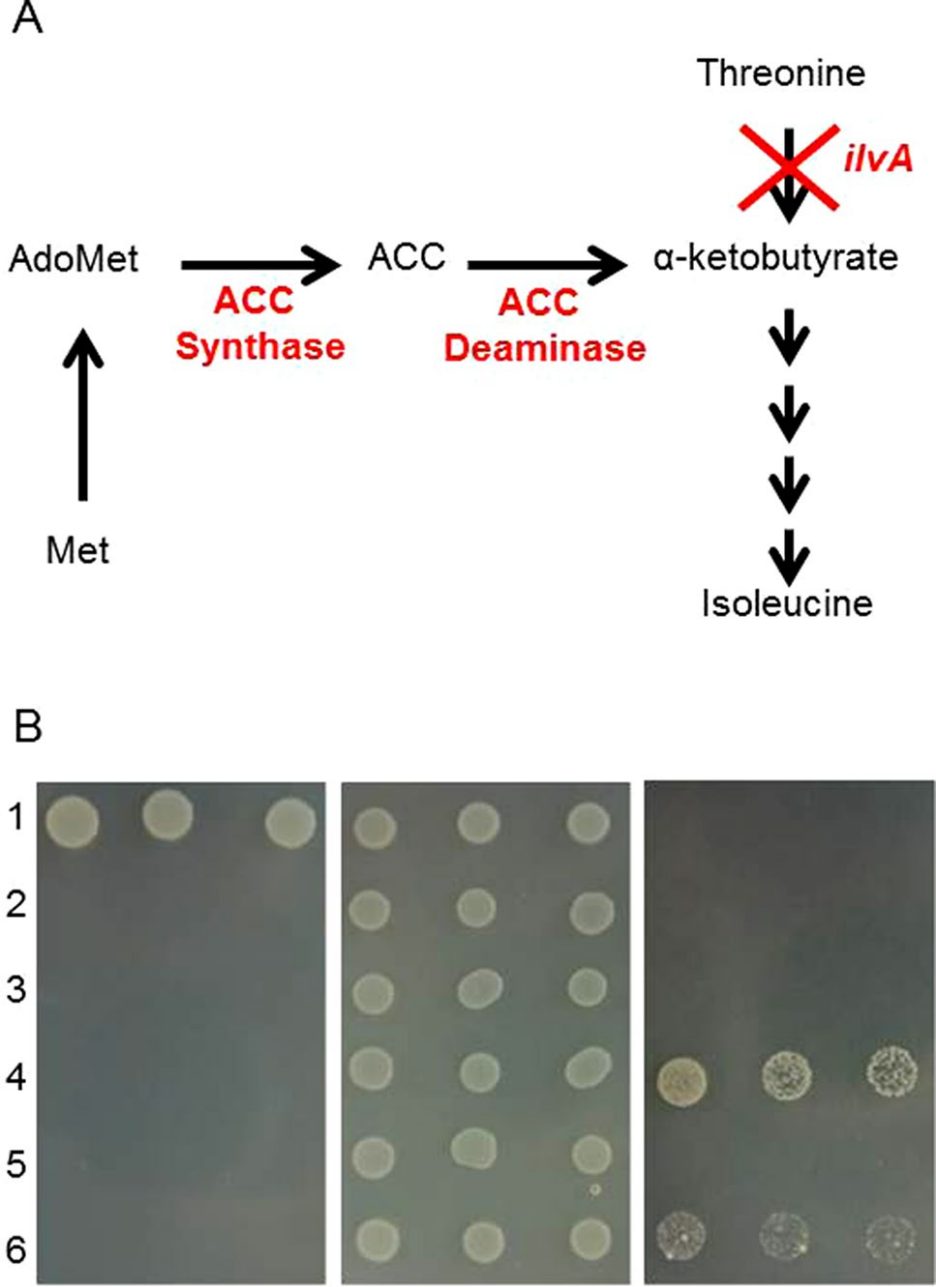
*In vivo* testing of 1-aminocyclopropane carboxylic acid (ACC) deaminase candidate genes. **(A)** schematic of modified isoleucine biosynthesis pathway. **(B)** suppression of isoleucine auxotrophy of the Δ*ilvA* mutant strain by expression of ACC deaminase genes. The Δ*ilvA* mutant of T7 express was transformed with the constructs indicated below. Cultures of the transformants and of the parental strains were spotted in serial dilutions (1:10, 1:50, 1:100, 1:200 from left to right) onto the following media: M9 (left panel), M9 + isoleucine (middle) and M9 + 0.05 mM ACC + 1 mM IPTG + 100 mg/L ampicillin (right). 1, T7 express; 2, T7 express Δ*ilvA*; 3, Δ*ilvA* + pET-duet1; 4, Δ*ilvA* + *ACD1* from *P. putida*; 5, Δ*ilvA* + *ACD1* from *F. graminearum*; 6, Δ*ilv5* + *ACD2* from *F. graminearum*.

To test for ACC formation activity, we cloned all three *ACS* candidate genes into pETDuet-1. This vector allows co-expression with an ACC deaminase in an *in vivo* test (see below). *E. coli* transformants were induced to express the *Fusarium* *ACS* candidate genes. The protein extracts were analysed by SDS-PAGE and bands of the expected sizes were detected (see **Supplementary Figure 5**). Feeding assays were performed with induced cells, and the consumption of methionine added to the medium, as well as the potential formation of ACC in the cell pellets were measured by GC-MS. Yet, none of the transformants showed activity. Similarly, protein extracts generated from induced ACS-expressing *E. coli* were tested using S-adenosyl-L-methionine as substrate, but again, ACC formation could not be detected. Some ACC synthases have been reported to be quite unstable proteins and difficult to handle. We therefore purified the proteins with spin columns to speed up the purification process, again without success. Consequently, we tried an *in vivo* approach by complementation of an isoleucine deficiency in *E. coli via *coupled ACS/ACD activity. The test was based on the ability of an *ilvA* mutant to grow on minimal medium (M9) without added isoleucine (Tarun et al., 1998), first due to the conversion of S-adenosylmethionine into ACC by an active ACS, and further conversion of ACC to the isoleucine precursor α-ketobutyrate by an ACC deaminase (see **Figure 1A**). For these experiments, the *Pseudomonas* ACD was cloned into pACYCDuet-1, which is compatible with pETDuet-1 and allows both proteins to be produced in one cell. We also combined the two genes in one plasmid (pETDuet-1), but no growth could be detected after co-expression, indicating a lack of ACS activity. Tests with ACC added to the medium and expression of ACD showed the functionality of the ACD part of the assay (see below).

### Testing ACD Candidate Genes in the *E. coli ilvA* Knock-out Strain

The product of the ACC deaminase reaction, α-ketobutyrate, is an intermediate of isoleucine biosynthesis in *E. coli*. If ACC is supplemented and ACD activity is present, an *ilvA* knockout strain is able to grow using the KBA formed from ACC to produce isoleucine. To allow testing of expression vectors based on the T7 RNA polymerase expression system (pET vectors), the *ilvA* mutation was introduced into the *E. coli* strain T7 Express by transferring the *ilvA*::Kan^R^ mutation from a strain from the *E. coli* knockout collection into the expression host (see *Materials and Methods* section). The ACD expression plasmids were introduced into the strain generated (T7 Express *ilvA*::Kan^R^). Both the pETDuet-1 vector containing the *Pseudomonas putida* ACD gene serving as positive control and the *Fusarium* *ACD2* expression vector allowed growth of the *ilvA *mutant on minimal media supplemented with ACC, demonstrating the presence of active enzymes. In contrast, no evidence for activity of the *ACD1* encoded protein was obtained with this assay (**Figure 1B**).

### Characterization of the ACC Deaminase Activity of the *ACD2* Gene Product

**Figure 2:**
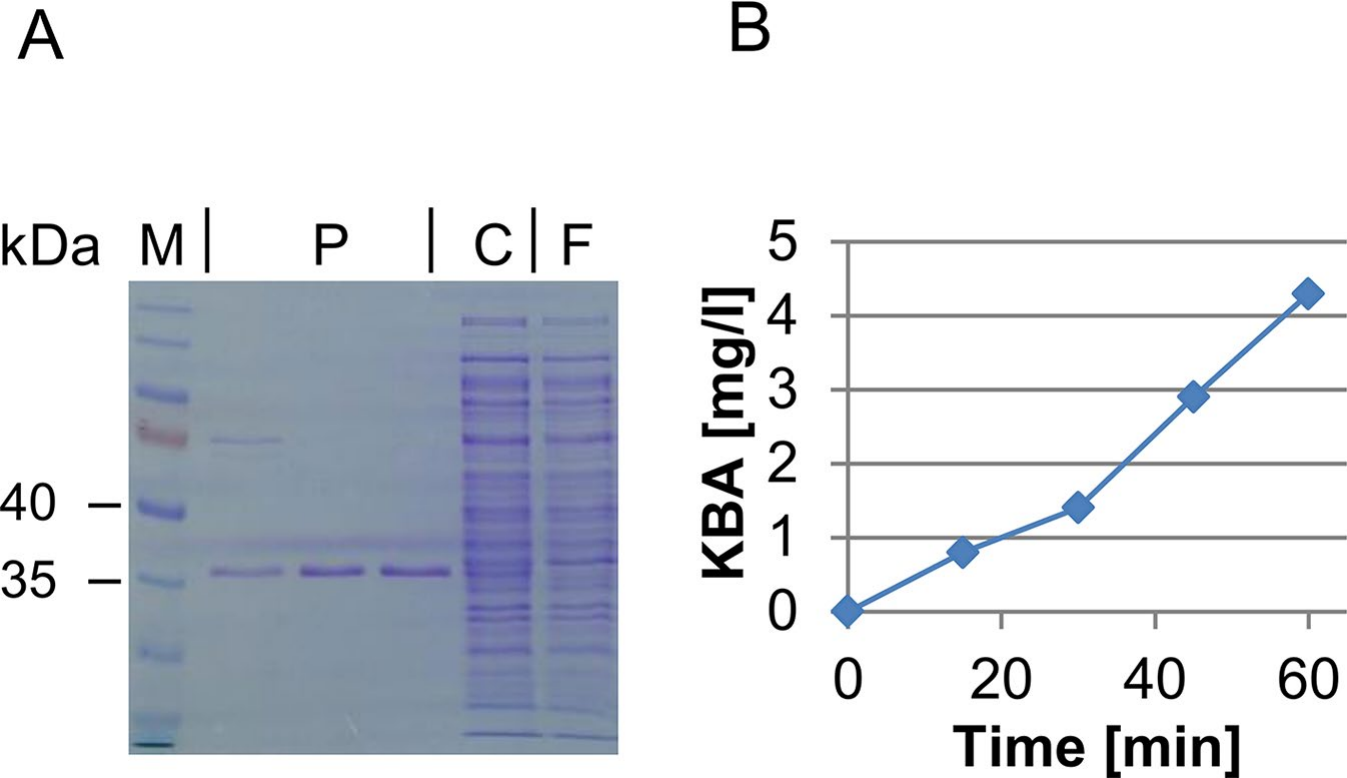
Purification and activity test of Acd2.** (A)** SDS-PAGE showing purification of Acd2 protein. M, size marker (Page Ruler, prestained, Thermo Scientific 26616); P, fractions of affinity purified Acd2; C, crude extract; F, flow through after affinity purification. **(B)** KBA formation by purified Acd2 protein over time; KBA concentration was determined by GC-MS as described in the *Materials and Methods* section.

The pilot experiment with the modified *E. coli* *ilvA* strain already indicated that Acd2 has the predicted ACC deaminase activity. This was also confirmed by a colorimetric assay with permeabilized cells (see **Supplementary Figure 6**). Furthermore, a test of the crude protein extract revealed that Acd2 shows ACC deaminase activity similar to the positive control of *Pseudomonas putida,* while Acd1 did not show measurable activity. Likewise, GC-MS analysis did not reveal activity of Acd1, but clearly KBA formation was detected with Acd2. A feeding assay using ACD-expressing *E. coli* revealed that independently of the expressed gene, supplemented KBA was consumed by *E. coli* and also partly transaminated to 2-aminobutyric acid released to the medium. The activity test was repeated with the affinity purified protein (**Figure 2A**). The Acd2 protein remained constantly active over the whole assay period observed (60 min) and was used to determine the enzymatic properties (**Figure 2B**). The K_m_ value of Acd2 for ACC was determined to be 3.3 ± 0.7 mM and v_max_ to 1.3 ± 0.06 µmol mg^−1^ min^−1^.

### Acd1 Shows d-Cysteine Desulfhydrase Activity

It had previously been shown in an elegant work, that ACC deaminases and d-cysteine desulfhydrases from *P. putida* not only share a high sequence similarity, but can be interconverted into each other by site directed mutagenesis (Todorovic and Glick, 2008).

**Figure 3:**
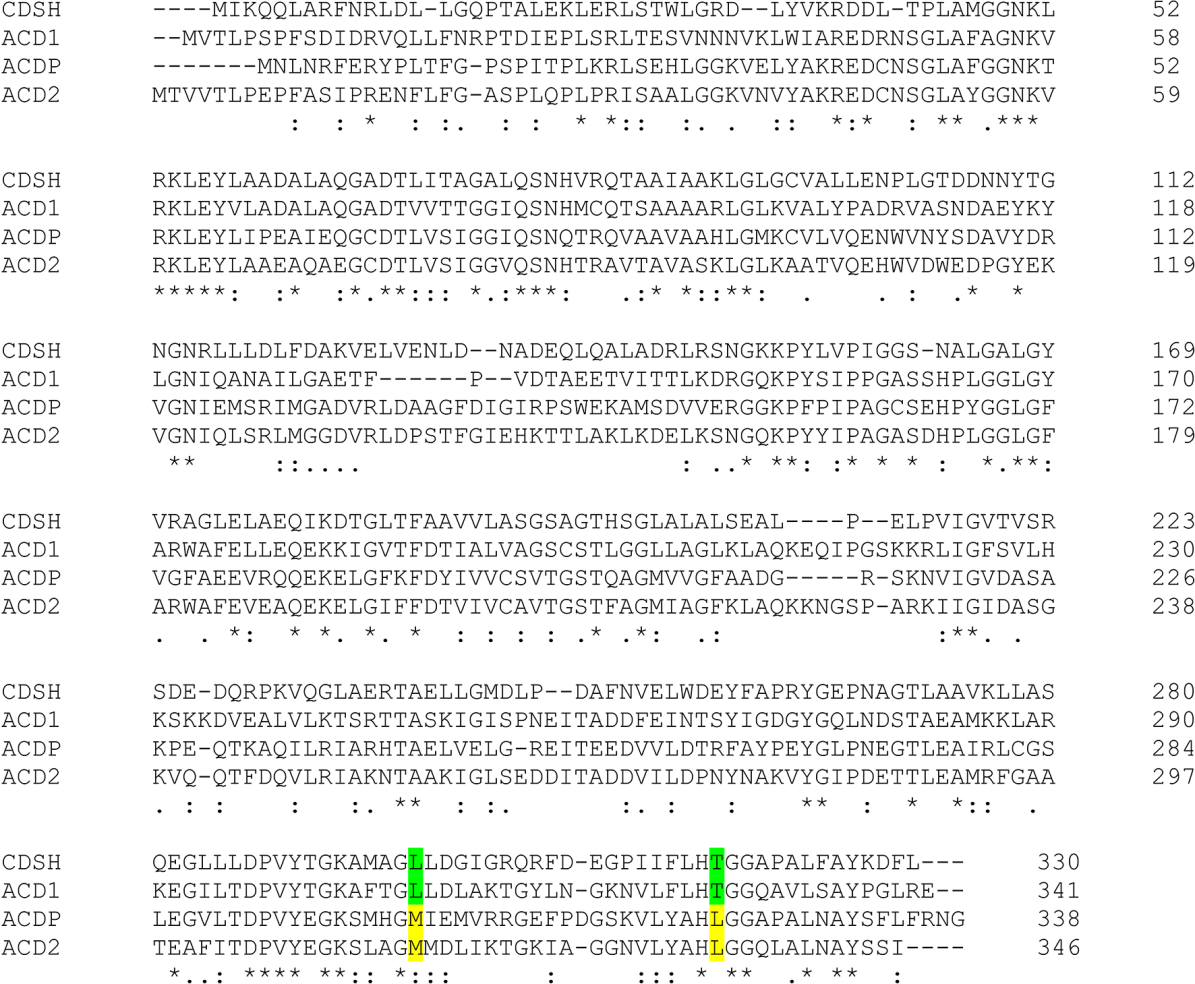
Alignment of D-cysteine desulfhydrase (CDSH) from *P. putida* (GenBank: AFY17401.1) and ACD from *P. putida* (ACDP; UniProtKB/Swiss-Prot: Q5PWZ8.1) with the predicted proteins Acd1 and Acd2 from *F. graminearum*. The highlighted amino acids are characteristic for a d-cysteine desulfhydrase (green) or for an ACC deaminase (yellow), respectively.

An alignment of both *Fusarium* candidate proteins with these *Pseudomonas* proteins is shown in **Figure 3**. The highlighted amino acids at positions 302 and 322 are specific for ACC deaminases and d-cysteine desulfhydrases. While Acd1 and the cysteine desulfhydrase from *P. putida* contain leucine and threonine, respectively, at these positions, Acd2 and the *Pseudomonas* ACC deaminase have methionine and leucine. Acd1 was therefore tested with d-cysteine as substrate. d-cysteine desulfhydrases convert d-cysteine into pyruvate, H_2_S and NH_3_. The formed pyruvate reacts with 2,4-dinitrophenylhydrazine, and this activity could be confirmed using the colorimetric assay. Neither l-cysteine nor S-(3-aminoethyl)-l-cysteine, which were used as alternative substrates, were consumed confirming the substrate specificity. The K_m_ value of Acd1 for d-cysteine was determined to be about 18 mM and v_max_ was 5.5 µmol mg^−^1 min^−^1. Since the ACD1/FGSG_02678 gene product shows d-cysteine desulfhydrase activity but not ACC deaminase activity we renamed the gene *DCS1*.

Due to the high specificity of the d-cysteine desulfhydrase and the presumably very low concentrations of d-cysteine *in planta*, we assumed that the fungus itself might be able to racemize l-cysteine to d-cysteine instead of obtaining the latter from the plant. To test this, a protein extract from a *Fusarium* culture, which had been pre-treated with d-cysteine, was incubated with l-cysteine for 24 h. Indeed, an increase in the d-cysteine concentration from a level below the limit of detection to around 2.5 mM was determined (data not shown, see Materials and Methods section) indicating the presence of a cysteine racemase. Yet, protein extracts of *Fusarium* cultures, which had been pre-exposed to L-cysteine first, did not show any detectable d-cysteine formation.

### *ACD2* and *DCS1* Knock-Out Strains Show Unaltered Virulence

**Figure 4:**
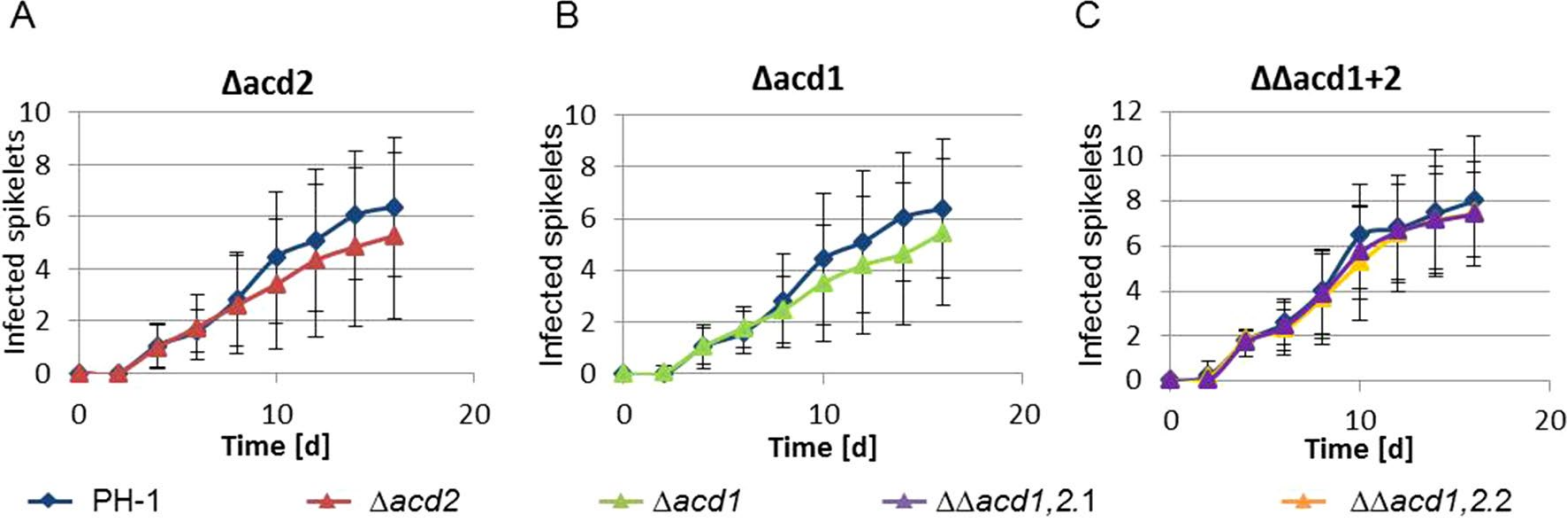
Virulence tests of the single and double knock-out mutants. Ears of the wheat cultivar Apogee were infected with PH-1 and the indicated mutant strains. **(A)** and **(B)**: PH-1 vs. Δ*acd2* and Δ*acd1*, respectively; the mean number of infected spikelets from 15 inoculated ears are shown for each strain and time point. **(C)** PH-1 (mean number of infected spikelets from 10 inoculated ears) compared to six independent double knock-out strains (ΔΔ*acd1,2*) derived from two independent acd1 single knock-out strains. For each graph, the mean number of infected spikelets from 10 inoculated ears for each of the three independent double knock-out strains were used at each time point. t-test was performed but no statistically significant difference was evidenced.

We constructed *acd2*Δ and *dcs1*Δ knock-out strains using a *hph-amdS* cassette flanked by two *loxP* sites (Steiger et al., 2011). The virulence of three independent mutants lacking *DCS1* and four independent *ACD2* deletion strains (**Supplementary Figures 7A** and **B**) was tested with 15 replicates per strain. On average, a marginally lower virulence of both, the *acd2*Δ and *dcs1*Δ knock-out strains was observed, but the results did not reach the threshold of statistical significance (**Figures 4A, B**). Virulence tests were also carried out with the double-knockout strains. Neither the single (*acd2*Δ or *dcs1*Δ), nor the double knock-out (*acd2*Δ* dcs1*Δ) strains (**Supplementary Figure 7C**) showed a significantly reduced virulence, indicating that these genes are dispensable during infection (**Figure 4C**).

The main problem with *F. graminearum* is contamination of grain with the mycotoxin deoxynivalenol (DON) and the plant detoxification product DON-3-glucoside (D3G). The *Arabidopsis* DON-detoxifying glucosyltransferase UGT73C5 is rapidly and transiently induced by ACC and jasmonic acid (Poppenberger et al., 2003). We therefore determined the concentrations of DON and D3G in infected ears at the endpoint by grinding and extracting 15 infected ears individually. The average DON values of the heads infected with *acd1*Δ were lower compared to wild type treated ears. Yet, these values were not significantly different due to high fluctuations in DON content between individual samples (**Supplementary Figure 8**).

### *Fusarium graminearum* Is Able to Use ACC as Sole Nitrogen Source

**Figure 5:**
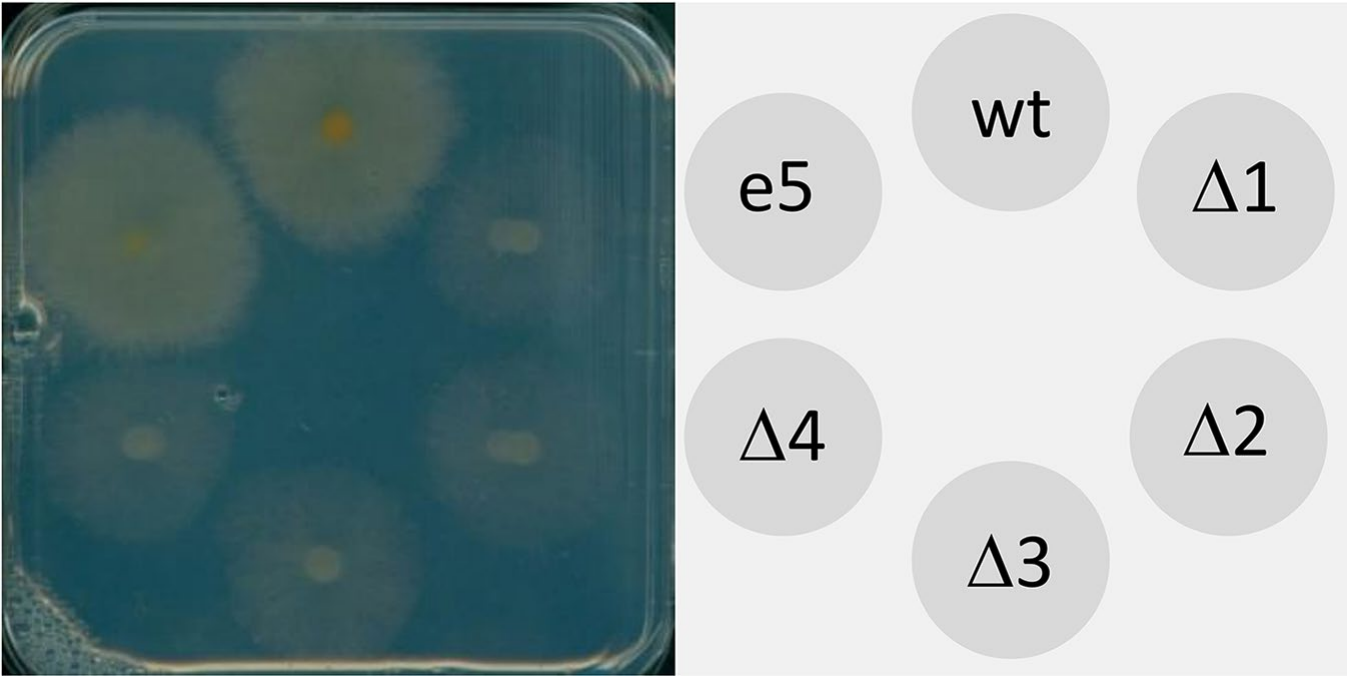
Utilization of 1-aminocyclopropane carboxylic acid (ACC) by *Fusarium graminearum*. Spores (10^5^) of the following strains were spotted onto modified FMM with ACC as sole nitrogen source: wt, PH-1; Δ, four independent *acd2*Δ knock-out strains; e, ectopic transformant. The plate was incubated at 20°C for 10 days in the dark.

Plant growth promoting rhizobacteria (PGPR), which provide increased stress tolerance to plants, had been shown to be able to grow on media with ACC as sole nitrogen source (Penrose and Glick, 2003). We therefore tested whether also *F. graminearum* can grow on ACC. Spores of four independent *acd2*Δ knockout strains were spotted on modified FMM with ACC as sole nitrogen source together with one ectopic mutant and the wild type control (PH-1). After 1 week of incubation, the wild-type and the ectopic mutant grew well on ACC as sole nitrogen source, while growth of the *ACD2* knockout strains was strongly retarded but not completely blocked (**Figure 5**). In contrast, all strains were equally well growing on standard FMM plates which were used as control (not shown). A possible explanation for continued growth of the mutant strains might be a partial non-enzymatic degradation of ACC in the medium.

## Discussion

Our bioinformatical investigation revealed that various *Fusarium *species (particularly from the *F. oxysporum* and *F. fujikuroi* complex) might possess EFE as a means to synthesize ethylene. Yet, it remains to be tested whether the candidate genes are indeed encoding enzymatically active proteins. Existing gene models have to be used with caution. For instance, in the case of *Fusarium mangiferae*, where fungal ethylene production has been implicated in malformation of mango fruits (Ansari et al., 2013), the gene model is questionable (see **Supplementary Figure 1**). A closer look at the DNA sequence of FMAN_06358 showed that the annotated intron might not be real. However, intron retention leads to a model with a frameshift, as indicated in **Supplementary Figure 1**. Questionable non-functional splice sites might be a cause for truncated *F. oxysporum* models lacking the last exon, and hence, for these species, experimental testing is warranted. Nevertheless, the focus of this study was on the cereal pathogen *F. graminearum*, and for this species, ethylene synthesis *via* the EFE pathway can be excluded.

Three candidates for ACC synthases were found in *F. graminearum*. Orthologs of these genes also exist in other *Fusarium* species. Therefore, certain *Fusarium* species apart from *F. graminearum *might potentially possess both the EFE and the ACS pathway, a phenomenon so far only described for *Penicillium digitatum* (Chalutz and Lieberman, 1977). Activity of heterologously expressed ACC synthases in *E. coli*, e.g. of the wheat ACC gene (Subramaniam et al., 1996, shown in the alignment in **Supplementary Figure 3**), has been described. Yet, many plant ACC synthases are notoriously unstable and difficult to handle *in vitro*. Our efforts to demonstrate synthesis of ACC with S-adenosyl-methionine *in vitro* with the *E. coli*-expressed affinity-purified protein were unsuccessful with all three candidates. Likewise, upon feeding of IPTG induced *E. coli* cells with methionine, no ACC formation in cell extracts could be detected. We therefore adopted the growth-based assay originally described by Tarun et al. (1998) for use with the T7 expression system. With the functional *Pseudomonas putida* ACC deaminase as a bridge (see **Figure 1**), no growth upon expression of any of the *F. graminearum* ACS candidate genes was observed on M9 medium (data not shown). However, in an experiment using headspace GC-MS, ethylene production by *F. graminearum* on minimal media supplemented with 20 mM methionine was observed, which means the fungus either has ACC synthase activity or uses the transamination pathway *via* KMBA.

Potentially, the *Fusarium* ACC synthases may be active only in a complex with other proteins and could play a role in the synthesis of secondary metabolites containing ACC or ACC-like substances. Recently, a first bacterial ACC synthase was described (Xu et al., 2018), which is involved in synthesis of guangnanmycin. Coronatine, a well-known *Pseudomonas* metabolite interfering with defense signaling (Geng et al., 2014), contains coronamic acid, which has strong structural similarity to ACC and induces the synthesis of ethylene from methionine (Kenyon and Turner, 1992) by activating JA signaling (Uppalapati et al., 2005). An isolate of the fungus *Trichothecium crotocinigenum*, belonging to the hypocreales like *Fusarium*, is able to produce a diketopiperazine type cyclic “dipeptide” containing ACC (cyclo-(L-pipecolinylaminocyclopropane-carboxylic acid) (Long, 2014)). Also in the cotoxin II nonribosomal peptide from the plant pathogen *Bipolaris zeicola* (Ueda et al., 1992) ACC is a building block. Yet, none of the ACC synthase candidate genes is located in or near a predicted gene cluster suggestive of a role in secondary metabolite biosynthesis. Both ACC deaminase genes and two of the three ACC synthase candidates are located on the slowly evolving, conserved sub-genome (Wang et al., 2017). Only *ACS2* (FGSG_07606) is located on the fast evolving subgenome. Analysis of published transcriptomics data from Zhang et al. (2012) and Wang et al. (2017) indicates that both *ACD1* (renamed *DCS1*) and *ACD2* are preferentially expressed during infection as compared to vegetative mycelial growth (5.3-fold and 6.8-fold higher, respectively), while of the ACC synthase genes, only the first candidate *ACS1* (FGSG_05184) exhibits increased expression in planta (3.8-fold). We plan in further work to perform metabolomic analyses using the generated ACS knockout strains. While this research was going on, an alternative gene model for ACS3 has been deposited in FUNGI DB. The former ACS3, FGSG_13587 gene model was modified upon availability of more robust RNA sequencing data. The current model, FGRAMPH1_01G27057, incorporates some changes due to an updated splicing pattern and extended N- and C-termini (**Supplementary Figure 3**). Furthermore, extensive adenosine to inosine editing (Bian et al., 2019) during sexual development was found in all three ACS candidate genes (Liu et al., 2019).

Regarding ACC oxidases, we are not aware of any biochemically characterized functional enzyme from an ascomycete. There are reports that ethylene synthesis and signalling play a role in sexual development of the slime mold *Dictyostelium discoideum* and also in the basidiomycete *Agaricus bisporus* (Wood and Hammond, 1977; Zhang et al., 2016). Silencing and overexpression of a putative *ACO* gene (http://dictybase.org/gene/DDB_G0277497) from the slime mold affected ethylene production (Amagai et al., 2007). Using the *D. discoideum* *ACO* gene as a BLAST query against the *F. graminearum *genome yields a list of genes that is similar to the results of a query using the *A. bisporus ACO*, but with a different order. Since we could not demonstrate ACC synthase activity, we did not further investigate whether these multiple candidates have indeed ACO activity, but this is clearly a topic for further research.

The search for potential ethylene production genes was inspired by the claim by Chen et al. (2009) suggesting *F. graminearum* might exploit ethylene production to trigger increased plant susceptibility. Yet, many transcriptome studies suggest the opposite (Kazan and Gardiner, 2018). This is true also for other *Fusarium* species. For instance, upregulation of banana ethylene biosynthesis and of ethylene-responsive transcription factors were implicated in the resistance response against *Fusarium oxysporum* f. sp. *cubense* (Li et al., 2013). Consequently, we also searched for ACC deaminase candidate genes having the potential to counteract ethylene defense signaling. Only one of the two purified ACD candidate gene products, Acd2, showed activity with ACC in the classical colorimetric assay with toluene-permeabilized cells (Penrose and Glick, 2003). The *F. graminearum* Acd2 enzyme has kinetic properties very similar to previously described ACDs from plant-beneficial bacteria or pathogenic fungi. For instance, the ACC deaminases from *Pseudomonas putida* and *Penicillium citrinum* showed K_m_ values of 3.40 and 4.80 mM, respectively, compared to 3.31 mM for *F. graminearum* Acd2. The enzymatic activity of Acd2 did allow growth of isoleucine-deficient *E. coli *on M9-medium supplemented with ACC. Yet, the *Fusarium* Acd2 protein seems to work better at lower temperatures, e.g., 30°C, than the routine *E. coli *growth temperature of 37°C.

With an increasing number of genome sequences available, it became evident that gene products from bacteria (Ekimova et al., 2018) and from plants with high similarity to ACC deaminases exist. However, they are inactive with ACC, but have d-cysteine desulfhydrase activity (DCS). It was shown by site-directed mutagenesis that exchanging only two amino acids was sufficient to convert *Pseudomonas* ACC deaminase into an enzyme with dcysteine desulfhydrase activity, and *vice versa* (Todorovic and Glick, 2008). We noticed that the inactive ACD1 (DCS1) candidate had the amino acids suggesting it might be a d-cysteine desulfhydrase (**Figure 3**). This was confirmed by enzymatic assays with the purified protein. The *F. graminearum* enzyme is inactive with l-cysteine. d-cysteine desulfhydrase catalyses the following reaction: d-cysteine + H_2_O → H_2_S + NH_3_ + pyruvate. The product H_2_S is, like ethylene, a gaseous signaling molecule with an increasingly recognized importance in animals and plants (Li et al., 2016; Filipovic and Jovanovic, 2017; Ziogas et al., 2018; Corpas et al., 2019). In crosstalk with other hormones, H2S is mediating increased resistance to abiotic stresses (Shi et al., 2015), for instance drought resistance in wheat (Li et al., 2017). In banana, hydrogen sulfide slows down senescence *via* affecting ethylene signaling (Ge et al., 2017) by downregulating the expression of both ACC synthase and ACC oxidase genes. Hydrogen sulfide can modify many proteins and change their activity by persulfidation or S-sulfhydration, where the cysteine-SH is converted into a persulfide group (-SSH) (Aroca et al., 2015). It has been recently shown that H_2_S in tomato negatively regulates ethylene biosynthesis by persulfidation of ACC oxidase (Jia et al., 2018). The relevance of H_2_S in plant-pathogen interaction is still largely unknown with the exception of some storage diseases (Huo et al., 2018).

*F. graminearum* and other species are able to produce auxin, and auxin levels are increased in *Fusarium* infected plants (Kidd et al., 2011; Wang et al., 2018). Elevated auxin levels trigger induction of ACC synthase and oxidase in various plants (Grossmann, 2000). We have unpublished evidence that, in an early stage of the interaction, auxin production is indeed a virulence factor of *F. graminearum* (Svoboda et al., in preparation). We hypothesized that ethylene might increase resistance of the plant when formed later in a necrotrophic interaction, and that the ACC deaminase candidate genes might be used by *Fusarium* to counteract ethylene formation. In this case, inactivation of ACC deaminase should reduce the virulence of mutants. To test the relevance of the ACC deaminase *ACD2* and *DCS1* (formerly *ACD1*) in plant disease development we generated single-gene knockout strains, and additionally the *acd2*Δ *dcs1*Δ double mutants. These strains were used for infection of the rapid cycling and highly *Fusarium*-susceptible wheat cultivar Apogee. No significant difference was detectable at the end-point or in the area under the disease progression curve. Furthermore, the levels of the mycotoxin DON and the plant detoxification product DON-3-O-glucoside determined at the endpoint of the infections after 16 days were highly variable. No significant difference was observed between wild type, the single *acd2*Δ and *dcs1*Δ strains, the ectopic insertion mutants or the double mutant. ACC deaminase and d-cysteine desulfhydrase are obviously dispensable for full virulence of *F. graminearum* on wheat, at least on the highly susceptible cultivar Apogee. This is in contrast to the interaction of tomato with the root-infecting pathogen *Verticillium dahliae*. In this pathosystem, host resistance to the wilt pathogen could be increased *via* expression of a bacterial ACC deaminase in tomato, which was under control of promoters limiting the expression to the site of infection (Robison et al., 2001). In line with this, it was recently reported that ACC deaminase knockout mutants of *Verticillium* were less virulent, while overexpression led to increased virulence (Tsolakidou et al., 2018). The only other evidence for a role of a fungal ACD we are aware of is the case of the plant growth promoting fungus *Trichoderma asperellum* T203. In this case, ACD was not deleted, but silenced using RNAi, the effects of which are often ephemeral. Nevertheless, the results suggested that the strain with reduced ACD activity has a decreased ability to promote root elongation of *Brassica napus* (Viterbo et al., 2010).

We tested whether *F. graminearum* can utilize ACC as a nitrogen source, which is clearly the case (**Figure 5**). Thus, instead of preventing the mobile signaling molecule ACC, generated at the infection front, from moving to other plant parts and triggering resistance, the main but marginal function of ACC deaminase could be utilization of a low-abundance nitrogen source. The enzyme has a K_m_ in the mM range. The highest reported (Wang et al., 2018) ACC concentration in infected ears of the highly *Fusarium* resistant cultivar Sumai3 of around 45 mg/kg could be relevant, especially assuming the local concentrations might be higher than this average. On the other hand, these <0.5 mM amounts of ACC seem to be of minor relevance, considering that reported levels of free amino acids in developing grain are in the range of 300 to 50 mM from 7 days post-anthesis to ripening (Howarth et al., 2008).

Likewise, the role of d-cysteine desulfhydrase in *Fusarium* is enigmatic. The expression of both *ACD2* and *DCS1* is higher during plant infection than in axenic cultures. A cysteine desulfhydrase gene (dcyD/yedO) also exists in *E. coli*. It is upregulated during sulfur starvation and may allow utilization of d-cysteine (if available in the environment). The only other known function of dcyD is protection against toxicity of very high levels of d-cysteine when present in minimal medium, since d-cysteine blocks threonine deaminase, the gene product of *ilvA* (Soutourina et al., 2001). Toxicity of d-cysteine is low in rich medium or when isoleucine is supplemented. Nevertheless, there are d-cysteine containing secondary metabolites that are orders of magnitude more toxic. If *Fusarium* is able to degrade them, *DCS1* might play a role to utilize the released d-cysteine. For instance, the *Aspergillus* metabolite malformin A contains two d-cysteine residues in the cyclic pentapeptide. Recently, malformin E was described to have a minimal inhibitory concentration of about 7 µM for *Fusarium solani* (Ma et al., 2016) and below 1 µM for *E. coli*. Many microbes can also produce highly toxic aminovinyl-cysteine-containing peptides that contain d-cysteine (Sit et al., 2011). This suggests a role in microbe-microbe interaction rather than in plant-pathogen interaction. Yet, the advantage seems minimal, since a *dcs1Δ* mutant is still able to grow on d-cysteine (data not shown), which is expected since the *F. graminearum* genome contains D-amino-acid oxidases.

We did not obtain evidence supporting a role of *DCS1* in plant disease. Still, utilization of a (marginal) sulfur source could be a function. The major sulfur transport forms during grain filling are glutathione and S-methyl-methionine (Bourgis et al., 1999), and both are highly abundant. While we are not aware of any reports of relevant concentrations of d-cysteine in plants, it has been described that peas possess transaminases producing d-alanine—and possibly other d-amino acids—in seedlings (Ogawa et al., 1973). It is, however, difficult to envision how a low-affinity enzyme with a K_m_ of ∼18 mM could make a relevant contribution to the utilization of sulfur sources. On the other hand, plants also have d-cysteine desulfhydrases (e.g. Arabidopsis AT1G48420), which may be involved in H_2_S signaling. Potentially, the fungus itself may be able to synthesize d-cysteine from l-cysteine. Our preliminary experiments indicated that *F. graminearum* has racemase activity that can potentially convert l-cysteine into d-cysteine. Cysteine racemase is a rarely reported activity (Espaillat et al., 2014). Formed d-cysteine might then be used by Dcs1 to produce a volatile H_2_S signal. Potentially, *F. graminearum* might be able to take up cysteine from the host and affect levels of glutathione, which is crucial for maintenance of the redox balance during infection. Glutathione (GSH) levels clearly have an impact on plant defense in general, most likely *via* affecting NPR1 and salicylic acid production (Ghanta et al., 2011; Kovacs et al., 2015) but also direct effects on ethylene production were reported (Datta et al., 2015). *Arabidopsis thaliana* transgenic plants with increased GSH content upregulated several ACS transcripts, and in addition, ACO protein levels were also increased, while in a *pad2-1* mutant with reduced GSH levels, the opposite effect was observed (Datta et al., 2015). Yet, our virulence tests do not support such a scenario. Inactivation of both ACC deaminase and d-cysteine desulfhydrase did not have a significant impact on the virulence of *F. graminearum*, at least not in the highly susceptible wheat cultivar Apogee. This outcome is not unprecedented. Other genes with a highly suggestive function were also found to be dispensable, such as genes encoding enzymes for degradation of salicylic acid (Hao et al., 2018; Qi et al., 2019; Rocheleau et al., 2019), despite clear evidence that salicylic acid plays an important role in the defense of wheat against *F. graminearum* (Makandar et al., 2006; Makandar et al., 2012). In future work we intend to investigate the role *Fusarium* ACC synthase candidate genes by generating triple knock-out mutants, to clarify the role of fungal ethylene production in the interaction with the host plant.

## Data Availability

All datasets generated for this study are included in the manuscript and the supplementary files.

## Author Contributions

TS constructed plasmids. TS performed experiments. GW, RS, and GA conceived the concept. GW supervised experimental work. KT and UG did the bioinformatical analyses. DS, RH, MV, and AP performed the analytical measurements. TS, KT, GW, and GA wrote the paper and all authors amended and corrected the paper.

## Funding

This work was funded by the Austria Science Fund (FWF), SFB Fusarium (F3702 and F3715), and the DFG (LAP3714).

## Conflict of Interest Statement

The authors declare that the research was conducted in the absence of any commercial or financial relationships that could be construed as a potential conflict of interest.

## Abbreviations

DON, deoxynivalenol; D3G, deoxynivalenol-3-O-glucoside; ACC, 1-aminocyclopropane carboxylic acid; KBA, ketobutyric acid; ACS, ACC synthase; ACD, ACC deaminase; ACO, ACC oxidase; EFE, ethylene forming enzyme; KMBA, 4-methylthio-2-oxobutanoic acid; CTR, constitutive triple response; EIN, ethylene insensitive; EIL, ethylene insensitive like; PR, pathogen related; FMM, fusarium minimal media.
