# Supplementary material for: Biochemical Characterization of the Fusarium graminearum Candidate ACC-Deaminases and Virulence Testing of Knockout Mutant Strains
Source: Front Plant Sci. 2019 Sep 10;10:1072. doi: 10.3389/fpls.2019.01072 (PMC6746940; doi:10.3389/fpls.2019.01072)
Supplement: Supplementary file 1 [file Table_1.docx]

Supplementary Table 1: Primer and fragment lengths for cloning of the candidate genes

| **ID** | **Primer sequences** | **Length [bp]** |
| --- | --- | --- |
| ACS1 | 5´- GAATTCAACACCAATGTCTCTTTCAA-3´  5´- GCGGCCGCTTACTATAGCAATGCATTAATCACCC-3´ | 1281 |
| ACS2 | 5´- GGATCCGATGACGCCGTCACACGCGAACCTT-3´  5´- GCGGCCGCTTAGCTGCGAAGGCTTGACCCATAAAGTGAAAC-3´ | 1365 |
| ACS3 exon1 | 5´- GGATCCGATGTTGATAAACTTACACTTCTC-3´  5´- TCCTCGTTTGAAATGGTAACATTTTCAGCTGTGCC-3´ | 132 |
| ACS3 exon2 | 5´- GGCACAGCTGAAAATGTTACCATTTCAAACGAGG-3´  5´- GGGGTAACACCGCCCAAGTGGGTTGTGGGGGTTG-3´ | 402 |
| ACS3 exon3 | 5´- CCCACAACCCACTTGGGCGGTGTTACCCCAAGTCC-3´  5´- GCGGCCGCTTACTACGATATTGCCATTTTG-3´ | 697 |
| ACD1 exon 1 | 5´- GGATCCAATGGTCACTCTTCCGTCAC -3´  5´- GCAGGATAAAGAGCGACTTTGAGGCCAAGTC -3´ | 303 |
| ACD1 exon 2 | 5´- ACTTGGCCTCAAAGTCGCTCTTTATCCTGC -3´  5´- GCGGCCGCTTACCGAATATGGGGCTACTCTCTTA -3´ | 725 |
| ACD2 exon 1 | 5´- GAATTCAATGACTGTCGTCACTCTTCCTG -3´  5´- TTCAGCGGCGAGGTACTCTAGCTTTCGGACCTTGTTTC -3´ | 187 |
| ACD2 exon 2 | 5´- GGTCCGAAAGCTAGAGTACCTCGCCGCTGAAG -3´  5´- GCGGCCGCGGCATTCTATTAACTTTCTATTTATCCTT -3´ | 892 |

Supplementary Table 2: Primer for amplification of the flanking regions for single knock-out strains

| **ID** | **Primer sequences** | **Length [bp]** |
| --- | --- | --- |
| ACS1 5´ | 5´-TAATGGCCGCATAGGCCTGGGTTGAACTTCGAAGAAG-3´  5´-AAACTAGTGGTGTTTAGTTGTCGTTGA-3´ | 510 |
| ACS1 3´ | 5´-AATGTCGACTGATTAATGCATTGCTATAG-3´  5´-ACAAGCTTCTGTCCCAACGTCTACTAAT-3´ | 441 |
| ACS2 5 | 5´-TAATGGCCGCATAGGCCGTTGCATTCTGCATCGA-3´  5´-AAACTAGTAGACTATCCAGGTTCTATCACG-3´ | 487 |
| ACS2 3´ | 5´-AATGTCGACAAATCGGCTGCGGTTG-3´  5´-ACAAGCTTCCTAAACCATATTACGTCGTC-3´ | 525 |
| ACS3 5´ | 5´-TAATGGCCGCATAGGCCAGAGGAACATTGTTTGTG-3´  5´-AAACTAGTGTCGACTGAGAAGTGTAAGTT-3 | 517 |
| ACS3 3´ | 5´-AATGTCGACTGGCCTTCGTCTCGGTGC-3´  5´-ACAAGCTTCGTAAAATCCCTCTCGCTC-3´ | 596 |
| ACD1 5´ | 5´-TAATGGCCGCATAGGCCCATTTCCATATTATTAACTT-3´  5´- AAACTAGTGATATCTTGTGTAAGTATGAAG-3´ | 520 |
| ACD1 3´ | 5´- CAGGCAAACGCCATTCTCG-3´  5´- AATTGCGGCCGCAAGCCGAGAGAACAGCCTG-3´ | 652 |
| ACD2 5´ | 5´- TAATGGCCGCATAGGCCACTAACCTTTAGAACAAT-3´  5´- AAACTAGTGGATATAGAAATTGATATAGA-3´ | 487 |
| ACD2 3´ | 5´- AATGTCGACTCTCGGTTTCGCGCGCT-3´  5´- ACAAGCTTGTGCCAATTGTCCTCCCAAAT-3´ | 491 |

Supplementary Table 3: Knock-out screening with multiplex PCR including scheme; A and B are the primers outside of the flanking region, primer C and D are located in the coding sequence of the respective gene and primer E and F are located in the resistance cassette; AB^R^ – antibiotic resistance; replacement of the gene leads to products with primers A+F and B+E

| **ID** | **Primer outside** | **Primer resistance cassette** | **Primer gene** | **Length [kb]** |
| --- | --- | --- | --- | --- |
| ACS1 5´ | 5´-GCTATCCTAAGCCTGCATC-3´ | 5´-AGAAGTACTCGCCGATAGTG-3´ | 5´-CCTCCCAGAGATCCATACC´-3´ | wt: 0.6  ∆: 1.4 |
| ACS1 3´ | 5´-GTTTCACAACTTGTAACGAGG-3´ | 5´-ACACCTGCCGTGTCAGCC-3´ | 5´-CGGATCATGTCACAGTCAAC-3´ | wt: 1.2  ∆: 0.8 |
| ACS2 5´ | 5´-GAGGCGGGAGCTGTTGGA-3´ | 5´-AGAAGTACTCGCCGATAGTG-3´ | 5´-ACGGGGGTTTTACATTTTGTG-3´ | wt: 0.7  ∆: 1.5 |
| ACS2 3´ | 5´-CTTGCTCAGGATTAATTATCAC-3´ | 5´-ACACCTGCCGTGTCAGCC-3´ | 5´-CTGCCGCGAGCATCCTC-3´ | wt: 0.6  ∆: 0.8 |
| ACS3 5´ | 5´-CCGGCGATCGAAGTTTTTGA-3´ | 5´-AGAAGTACTCGCCGATAGTG-3´ | 5´-CCGTTGCGATAGAGCACTC-3´ | wt: 0.6  ∆: 1.4 |
| ACS3 3´ | 5´-GAGGGCAACATCTACCTTGT-3´ | 5´-ACACCTGCCGTGTCAGCC-3´ | 5´- ACGTCACCTACGGACTAAGT -3´ | wt: 1.0  ∆: 1.3 |
| ACD1 5´ | 5´-CGTGGAGATTGAAAACAATGC-3´ | 5´-AGAAGTACTCGCCGATAGTG-3´ | 5´-GCGATTGAACAACAGCTGGA-3´ | wt: 0.6  ∆: 1.4 |
| ACD1 3´ | 5´-CAGCACAAGTCATACTTCAAC-3´ | 5´-ACACCTGCCGTGTCAGCC-3´ | 5´-TCACGACTCTTAAAGATCGTG-3´ | wt: 1.0  ∆: 1.3 |
| ACD2 5´ | 5´-CTACCGATGGGCCTTATTCT-3´ | 5´-AGAAGTACTCGCCGATAGTG-3´ | 5´-CTACATCCCAGCAGGTGC-3´ | wt: 0.6  ∆: 1.4 |
| ACD2 3´ | 5´-CAAGCCGGATATATACATCTC-3´ | 5´-ACACCTGCCGTGTCAGCC-3´ | 5´-AGAGGTGAAGCTCCAAAGAG-3´ | wt: 0.6  ∆: 0.8 |

3´ UTR

ACS/ ACD

5´ UTR

AB^R^

HSVtk

5´ UTR

3´ UTR

A

B

F

E

C

D

A

B

Supplementary Table 4: Strains and plasmids generated in this study

| pTS1 | FGSG_13587 (ACS3) in pETDuet-1 using BamHI and NotI |
| --- | --- |
| pTS2 | FGSG_02678 (ACD1) in pETDuet-1 using BamHI and NotI |
| pTS3 | FGSG_12669 (ACD2) in pETDuet-1 using EcoRI and NotI |
| pTS4 | FGSG_05184 (ACS1) in pETDuet-1 using EcoRI and NotI |
| pTS5 | FGSG_07606 (ACS2) in pETDuet-1 using BamHI and NotI |
| pTS11 | ACC-deaminase of *Pseudomonas putida* (2228), cloned from pET 30a (cut with BglII/HindIII) in pET-Duet1 (MCS1) in BamHI/HindIII site |
| pTS160 | ACC-deaminase from *P. putida* digested with BglII/HindIII and cloned in pACYC-DUET1 (BamHI/HindIII), Chloramphenicol resistance |
| T7 express ∆ilvA::KanR | *E. coli* T7 express ∆ilvA::KanR #1; Isoleucine biosynthesis disrupted --> not able to grow on M9 media without Ile |
| pTS14 | 5´flanking region of FGSG_05184 (ACS1) in pASB42 (BcuI/SfiI); |
| pTS24 | 3´flanking region of FGSG_05184 (ACS1) in pASB42 (HindIII/SalI); |
| pTS35 | pASB42 with 3´flanking region of FGSG 07606 (ACS2) cloned in HindIII/SalI site; |
| pTS45 | pASB42 with 3´flanking region of FGSG 13587 (ACS3) cloned in HindIII/SalI site; |
| pTS52 | pASB42 with 3´flanking region of FGSG 02678 (ACD1) cloned in HindIII/SalI site; |
| pTS58 | pASB42 with 5´flanking region of FGSG 07606 (ACS2) cloned in BcuI/SfiI site; |
| pTS61 | pASB42 with 5´flanking region of FGSG 13587 (ACS3) cloned in BcuI/SfiI site; |
| pTS64 | pASB42 with 5´flanking region of FGSG 02678 (ACD1) cloned in BcuI/SfiI site; |
| pTS67 | pASB42 with FGSG 12669 (ACD2) 5´flanking region, cloned in BcuI/SfiI site, |
| pTS72 | pASB42 with FGSG 12669 (ACD2) 3´flanking region, cloned in BsiWI/SalI site, |
| pTS189 | FGSG 12669 (ACD2 5´) flanking region cloned in pKT245 (BcuI/SfiI) |
| pTS190 | FGSG 12669 (ACD2 3´) flanking region cloned in pKT245 (NotI/SalI) |

Supplementary Table 5: Single knock-out strains generated in this study; the numbers in the brackets for the ACD1 knock out strains indicate the strains used for generating the double knock out mutants

| IATS1 | *acd2Δ::loxP-amdS-hph-loxP* | Knock out of FGSG 12669 (ACD2);HygR, |
| --- | --- | --- |
| IATS2 | *acd2Δ::loxP-amdS-hph-loxP* | Knock out of FGSG 12669 |
| IATS3 | *acd2Δ::loxP-amdS-hph-loxP* | Knock out of FGSG 12669 |
| IATS4 | *acd2Δ::loxP-amdS-hph-loxP* | Knock out of FGSG 12669 |
| IATS7 | *acd2Δ::loxP-amdS-hph-loxP* | Knock out of FGSG 12669 (ectopic) |
| IATS8 | *acd2Δ::loxP-amdS-hph-loxP* | Knock out of FGSG 12669 |
| IATS100 | *acs1Δ::loxP-amdS-hph-loxP* | Knock out of FGSG 05184 (ACS1) |
| IATS101 | *acs1Δ::loxP-amdS-hph-loxP* | Knock out of FGSG 05184 |
| IATS102 | *acs1Δ::loxP-amdS-hph-loxP* | Knock out of FGSG 05184 |
| IATS103 | *acs1Δ::loxP-amdS-hph-loxP* | Knock out of FGSG 05184 |
| IATS104 | *acs1Δ::loxP-amdS-hph-loxP* | Knock out of FGSG 05184 |
| IATS105 | *acs1Δ::loxP-amdS-hph-loxP* | Knock out of FGSG 05184 |
| IATS106 | *acs1Δ::loxP-amdS-hph-loxP* | Knock out of FGSG 05184 |
| IATS108 | *acs1Δ::loxP-amdS-hph-loxP* | Knock out of FGSG 05184 |
| IATS109 | *acs1Δ::loxP-amdS-hph-loxP* | Knock out of FGSG 05184 |
| IATS110 | *acs1Δ::loxP-amdS-hph-loxP* | Knock out of FGSG 05184 |
| TS_ACS2Δ_2 | *FGSG_07606Δ::loxP-amdS-hph-loxP* | Knock out of FGSG 07606 |
| TS_ACS2Δ_5 | *FGSG_07606Δ::loxP-amdS-hph-loxP* | Knock out of FGSG 07606 |
| TS_ACS2Δ_8 | *FGSG_07606Δ::loxP-amdS-hph-loxP* | Knock out of FGSG 07606 |
| TS_ACS2Δ_13 | *FGSG_07606Δ::loxP-amdS-hph-loxP* | Knock out of FGSG 07606 |
| TS_ACS2Δ_21 | *FGSG_07606Δ::loxP-amdS-hph-loxP* | Knock out of FGSG 07606 |
| TS_ACS2Δ_23 | *FGSG_07606Δ::loxP-amdS-hph-loxP* | Knock out of FGSG 07606 |
| TS_ACD1Δ_2 | *FGSG_02678Δ::loxP-amdS-hph-loxP* | Knock out of FGSG_02678 (#1351) |
| TS_ACD1Δ_4 | *FGSG_02678Δ::loxP-amdS-hph-loxP* | Knock out of FGSG_02678 |
| TS_ACD1Δ_12 | *FGSG_02678Δ::loxP-amdS-hph-loxP* | Knock out of FGSG_02678 |
| TS_ACD1Δ_13 | *FGSG_02678Δ::loxP-amdS-hph-loxP* | Knock out of FGSG_02678 (#1353) |
| TS_ACD1Δ_20 | *FGSG_02678Δ::loxP-amdS-hph-loxP* | Knock out of FGSG_02678 |
| TS_ACS3Δ_5 | *FGSG_13587Δ::loxP-hph-loxP* | Knock out of FGSG_13587 |
| TS_ACS3Δ_13 | *FGSG_13587Δ::loxP-hph-loxP* | Knock out of FGSG_13587 |
| TS_ACS3Δ_29 | *FGSG_13587Δ::loxP-hph-loxP* | Knock out of FGSG_13587 |
| TS_ACS3Δ_44 | *FGSG_13587Δ::loxP-hph-loxP* | Knock out of FGSG_13587 |
| TS_ACS3Δ_51 | *FGSG_13587Δ::loxP-hph-loxP* | Knock out of FGSG_13587 |

Supplementary Table 6: Double knock out strains generated in this study

| TS-1351 ΔACD2_5 | *FGSG_02678 Δ::loxP-hph-amdS-loxP FGSG_12669 Δ::loxP-nptII-HSVtk-loxP* |
| --- | --- |
| TS-1351 ΔACD2_29 | *FGSG_02678 Δ::loxP-hph-amdS-loxP FGSG_12669 Δ::loxP-nptII-HSVtk-loxP* |
| TS-1351 ΔACD2_33 | *FGSG_02678 Δ::loxP-hph-amdS-loxP FGSG_12669 Δ::loxP-nptII-HSVtk-loxP* |
| TS-1353 ΔACD2_2 | *FGSG_02678 Δ::loxP-hph-amdS-loxP FGSG_12669 Δ::loxP-nptII-HSVtk-loxP* |
| TS-1353 ΔACD2_21 | *FGSG_02678 Δ::loxP-hph-amdS-loxP FGSG_12669 Δ::loxP-nptII-HSVtk-loxP* |
| TS-1353 ΔACD2_31 | *FGSG_02678 Δ::loxP-hph-amdS-loxP FGSG_12669 Δ::loxP-nptII-HSVtk-loxP* |
