# Supplementary material for: Biochemical Characterization of the Fusarium graminearum Candidate ACC-Deaminases and Virulence Testing of Knockout Mutant Strains
Source: Front Plant Sci. 2019 Sep 10;10:1072. doi: 10.3389/fpls.2019.01072 (PMC6746940; doi:10.3389/fpls.2019.01072)
Supplement: Supplementary file 2 [file Table_2.docx]

P.syringae ------------------------------------------------------------ 0

P.chrysogenum MLSHRSVSIALRPPV-ICHNSIRHA-PN-LLARQQ-TKGLATTTAPSDLGSTMPPNYVAR 56

P.digitatum ----------------------------------------------------MPPNYVAR 8

P.digitatum_1 MFSHRSVSIALRPPG-LCHITFRQAYPI-LLTRQQ-TKSLTTTTAPSNLGSTMPPNYVAR 57

F.oxysporum ------MSLLNASPAAMKAISLPSTAFVPTAARSMATAATQPHPQGLEKASDVPPGHLAT 54

F.venenatum ------MTLLSSSRSITRTMALSSTTVQPIMAKNMATAATQPHAESLPNASGIPPGHTAT 54

F.avenaceum ------MSLLSSSRSITRTVALSSTIPHPVVSRNMATAAAQLHAQSLPDASGIPPGHAAT 54

F.mangiferae -------------------M---------ASAAAAVAPAPQPHSPTILNASGPPPGHSVT 32

F.mangiferae_1 -------------------M---------ASAAAAVAPAPQPHSPTILNASGPPPGHSVT 32

F.fujikuroi ---------------MVRKM---------ASAAAAAAPTPQPHSPTILNASGPPPGHSIT 36

P.syringae MTNLQTFELPTEVTGCAADISLGRALIQAWQKDGIFQIKTDSEQDRKTQEAMAASKQFCK 60

P.chrysogenum VGQLKTFTLPEKATGSPGDVEMGKALINAWREDGILQIAMNPKQQDLFNKAFAASKRFFA 116

P.digitatum VGQLKTFTLPETATGSPSDVELGKAMINAWREDGILQVSMSPRQQALFENASAASKRFFA 68

P.digitatum_1 VGQLKTFTLPETATGSPSDVELGKAMINAWREDGILQVSMSPRQQALFENASAASKRFFA 117

F.oxysporum TGKLQTFILPEEVSGSEEDKKLGKAIVDAWRKDGILQIAMKPEQEGLYKAANLASKRFFS 114

F.venenatum VGKLQTFVLPEKVSGSVEDKNLGKTIIDAWKKDGILQIAMKPEQQGLYKNANLASKRFFS 114

F.avenaceum VGNLQTFVLPEKVSGSAEDKKLGKAIIDAWKKDGILQIAMKPEQQGLYKAANLASKRFFS 114

F.mangiferae AGNLQTFILPDKVSDTEANHKHDTRAA--------------------------------- 59

F.mangiferae_1 AGNLQTFILPDKVSDTEANRKLGKALVEAWQKDGILQINMTPEQHSLYKSANYASRRFFS 92

F.fujikuroi VGNLQTFILPDKVSDTEANRKLGKALVEAWQKDGILQISMTPEQHSLYKSANYASRRFFS 96

:*:** ** .:. : . .

P.syringae EPLTFKSSCVSDLTYSGYVASGEEVTAGKPDFPEIFTVCKDLSVGDQRVKAGWPCHGPVP 120

P.chrysogenum LPPNVKANCVDTQSYAGYIASGEEITDGIADYSEIFTVTKDLPLEEPRVAAKWPCHGPCP 176

P.digitatum MPPNQKAACVDTQSYAGYIASGEEITDGIADYSEIFTVTKDLPLDEPRVEAKWPCHGPCP 128

P.digitatum_1 MPPNQKAACVDTQSYAGYIASGEEITDGIADYSEIFTVTKDLPLDEPRVEAKWPCHGPCP 177

F.oxysporum KPHAQKAACIDSQTYSGYIASGEEMTDGIADYSEIFTVTEDLELDEPRVVAKWPCHGRCP 174

F.venenatum KPYAQKSACVDSQTYSGYIASGEELTDGIADYSEIFTVTKDLELDEPRVVAKWPCHGRCP 174

F.avenaceum KPHAQKSACIDSQTYSGYIASGEELTDGIADYSEIFTVTKDLDLDEPRVVAKWPCHGRCP 174

F.mangiferae --FTLQKCQLRYQTYSGYIASGEELTDGIADYSEIFTVTKDLELDEPRVVAKWPCHGRCP 117

F.mangiferae_1 KPYAQKAACVDSQTYSGYIASGEELTDGIADYSEIFTVTKHLELDEPRVVAKWPCHGRCP 152

F.fujikuroi KPYAQKAACVDSQTYSGYIASGEELTDGIADYSEIFTVTKDLELDEPRVVAKWPCHGRCP 156

: : :*:**:*****:* * *: ***** :.* : : ** * ***** *

P.syringae WPNNTYQKSMKTFMEELGLAGERLLKLTALGFELPINTFTDLTRDGWHHMRVLR---FPP 177

P.chrysogenum WPDIDTKAPIQEYMDSLGSSGETLLQLIEHGLSLEPKTLTSLTKDGWHHLRTLR---FPQ 233

P.digitatum WPDVDMRTPIQQYMDSLGKSGETLLQMIEYGLSLHPDTLTSLTKDGWHHLRILR---FPQ 185

P.digitatum_1 WPDVDMRTPIQQYMDSLGKSGETLLQMIEYGLSLHPDTLTSLTKDGWHHLRILR---FPQ 234

F.oxysporum WPDYEMKNPMERYMNSLGESGEILLKLTELGLGVPEGSLTDYTKDGWHHMRILR---FPA 231

F.venenatum WPDYEMQNPMERYMNSLRESGETLLQLTELGLDVPAGSLTDYTKDGWHHMRILR---FPA 231

F.avenaceum WPDYEMQNPMERYMNSLGESGETLLKLTELGLDVPEGSLADYTKDGWHHMRILR---FPA 231

F.mangiferae WPDYEMQNPMHRYMQSLGGVGETLLQLTELGLGVPQGSLTNYTEDGWHHLRILRHGSFPA 177

F.mangiferae_1 WPDYEMQNPMHRYMQSLGGVGETLLQLTELGLGVPQGSLTNYTEDGWHHLRILR---FPA 209

F.fujikuroi WPDYEMQNPMHRYMKSLGGVGETLLQLTELGLGVPQGSLTNYTQDGWHHLRILR---FPA 213

**: : :. :*..* ** **:: *: : :::. *.*****:* ** **

P.syringae QTST-----LSRGIGAHTDYGLLVIAAQDDVGGLYIRPPVEGEKRNRNWLPGESSAGMFE 232

P.chrysogenum NNKTNGRGKEGRGIGSHTDYGLLVIAGQDEVGGLFIRPPYSDEKL-ENWK--SSAAGFRE 290

P.digitatum NNKTNGRGKKGRGIGSHTDYGLLVIAAQDEVGGLFIRPPADDEKL-ENWK--NSAAGFRE 242

P.digitatum_1 NNKTNGRGKKGRGIGSHTDYGLLVIAAQDEVGGLFIRPPADDEKL-ENWK--NSAAGFRE 291

F.oxysporum INKTNGKGKEGRGIGSHTDYGLLVLAAADDVGALLVRPPQQGDNF-SNWE--KSAAGFKE 288

F.venenatum INKTNGKGKEGRGIGSHTDYGLLVLAAADDVGALLVRPPQEGDDF-ANWE--KSAAGFKE 288

F.avenaceum MNKTNGKGKEGRGIGSHTDYGLLVLAAADDVGALLVRPPQKGDDF-ANWE--ASAAGFKE 288

F.mangiferae INKTNGKGKEGRGIGSHTDYGLLVIAAADDVGGK-------YSTH-FSLI--SRD----- 222

F.mangiferae_1 INKTNGKGKEGRGIGSHTDYGLLVIAAADDVGGN--------------------AAGFKE 249

F.fujikuroi INKTNGKGKEGRGIGSHTDYGLLVIAAADDVGGN-------DEKL-ANWE--SSAAGFKE 263

..* .****:********:*. *:**.

P.syringae HDEPWTFVTPTPGVWTVFPGDILQFMTGGQLLSTPHKVKLNTRERFACAYFHEPNFEASA 292

P.chrysogenum HDDRWTYVPPVPGVFTVFPGDMMQFMTNSYLPSTPHKVGLNTRERYAFAYFHEPSFQAEI 350

P.digitatum DDERWVYVPPVPGVFTVFPGDIMQFMTNSYLPSTPHKVGLNTRERFAFAYFHEPSFQAVV 302

P.digitatum_1 DDERWVYVPPVPGVFTVFPGDIMQFMTNSYLPSTPHKVGLNTRERFAFAYFHEPSFQAVV 351

F.oxysporum DDEGWLFVPPAENVFTVFPGDMMQYLTNSFLPSTPHKVGLNIRERFAFAYFHEPSFQAVI 348

F.venenatum DSEGWLFVPPAENVFTVFPGDMMQYLTNSYLPSTPHKVGLNHRERFAFAYFHEPSFQAVI 348

F.avenaceum DADGWLFVPPAKNVFTVFPGDMMQYLTNSFLPSTPHKVGLNHRERFAFAYFHEPSFQATI 348

F.mangiferae ------------------------------------------------------------ 222

F.mangiferae_1 DDERWIFVPPAEN-----------YITNSALPSTPHKVGLNLRERFAFAYFHEPSFQAVV 298

F.fujikuroi DDERWVFVPPAENVFT--------YITNSALPSTPHKVGLNLRERFAFAYFHEPSFQAVV 315

P.syringae YPLFEP----SANERIHYGEHFTNMFMRCYPDRITTQRINKENRLAHLEDLKKYSDTRAT 348

P.chrysogenum SPIAKLYDGKPPDEKNHYGTHFTNMFMRNYPDRVTTERILKEDRLKLLDLPELRTK---- 406

P.digitatum SPVAKLYDGQPPVEKIHYGTHFTNMFMRNYPDRITTERIIKEDRLQLLDRPELRTQ---- 358

P.digitatum_1 SPVAKLYDGQPPVEKIHYGTHFTNMFMRNYPDRITTERIIKEDRLQLLDRPELRTQ---- 407

F.oxysporum KPLPGYNAGQEPKEGVHYGKHFTDMFMRNYPERITTQRLLEEGRYDLLKQESLRTMSP-- 406

F.venenatum NPLPGYDAGQEPKEGVHYGKHFTDMFMRNYPQRITTQRLIDEGRYDLLKQESLYTMSP-- 406

F.avenaceum KPLPGYDAGQEPKEGVHYGKHFTDMFMRNYPQRITTQRLLDEGRYDLLKQDALHTMSP-- 406

F.mangiferae ------------------------------------------------------------ 222

F.mangiferae_1 KPLPGYDVGQEPKDGIHYGKHFTNMFMRNYPQRITTQRLNDEGRYRLLGQESLQTMAP-- 356

F.fujikuroi KPLPGYDVGQEPKDGIHYGKHFTNMFMRNYPQRITTQRLNDEGRYRLLEQESLQTMAP-- 373

P.syringae GS 350

P.chrysogenum -- 406

P.digitatum -- 358

P.digitatum_1 -- 407

F.oxysporum -- 406

F.venenatum -- 406

F.avenaceum -- 406

F.mangiferae -- 222

F.mangiferae_1 -- 356

F.fujikuroi -- 373

Supplementary Figure 1: Custal omega alignment of confirmed ethylene forming enzyme (EFE) from *Pseudomonas syringae* (UniProtKB/Swiss-Prot: P32021.1) and *Penicillium digitatum* (EKV19239.1) as well as a highly similar but non active gene of *Pseudomonas. chrysogenum* (XP_002562422.1) with similar genes from *F. oxysporum* (RKK90967.1), *F. venenatum* (XP_025583657.1), *F*. *avenaceum* (KIL86278.1), *F. fujikuroi* (XP_023429962.1) and *F. mangiferae* (CVK86342.1). *P. digitatum*_1 is the sequence of *P. digitatum* with a signal peptide at the N‑terminus encoding an amphiphatic α-helix motif encoding the mitochondrial localization signal. *F. mangiferae*_1 is an alternatively spliced version of the *F. mangiferae* sequence. However, the violet and grey highlighted regions were also annotated as an intron in the alternatively spliced version. Taking a closer look at the reading frames we identified a shift from frame 1 (purple) to reading frame 3 (light blue). Amino acids shaded in yellow when changed to the *P. chrysogenum* version inactivate the protein; Green shaded amino acids are predicted to bind Fe(II); Red amino acids are predicted to bind 2-oxoglutarate and correspond to EFE-siteI; Grey amino acids indicate putative mitochondrial leader sequences.


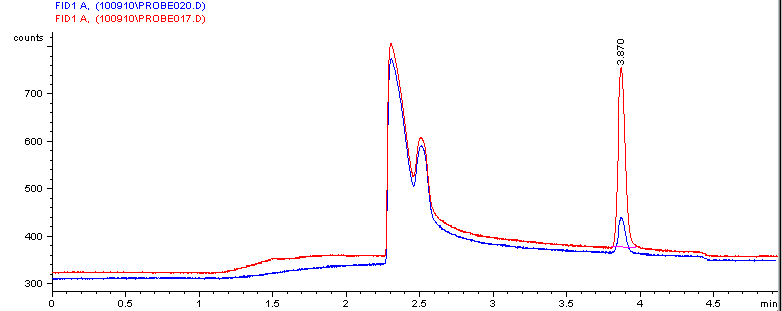


PDA + methionine

PDA

Supplementary Figure 2: GC-MS analysis of ethylene formation by *F. graminearum* grown on (blue) potato dextrose agar (PDA) and PDA supplemented with 20 mM methionine (red); elution of ethylene after 3.87 minutes

T.aestivum --------------MAAGDGHGENSSYFDGWKAYDMNPFHPQDNRGGVIQMGLAENQLSL 46

ACS3 -----------MSALSQRATEAVG-GLDLPWRFAPRGDYDPDHNPTGLISFGTAEN---- 44

ACS3_1 -----------MSALSQRATEAVG-GLDLPWRFAPRGDYDPDHNPTGLISFGTAEN---- 44

ACS3_FungiDB MLINLHFSVDKMSALSQRATEAVG-GLDLPWRFAPRGDYDPDHNPTGLISFGTAENALVT 59

ACS3_FungiDB_1 MLINLHFSVDKMSALSQRATEAVG-GLDLPWRFAPRGDYDPDHNPTGLISFGTAENALVT 59

ACS2 -------MTPSHANLSTRGELFASPAYRQSLLDILSDLWHPETKSSGYT--------LMH 45

ACS2_1 -------MTPSHANLSTRGELFASPAYRQSLLDILSDLWHPETKSSGYT--------LMH 45

P.citrinum ---------MTQSVLSRRAQDVVDAGSENPMWDVMKDTWHASTNPTGYVNVGVAENALMH 51

ACS1 ------------MSLSKRANDAEKAIEGMDLWEVIPNLFDQETNPDGIVSLGVAENTLMH 48

ACS1_1 ------------MSLSKRANDAEKAIEGMDLWEVIPNLFDQETNPDGIVSLGVAENTLMH 48

:: . :. . : *

T.aestivum DLIEEWSKAHPEAS---I-CTAEGA---SQFKRIANFQDYHGLPEFRQAMAQFMGQVRGW 99

ACS3 --------------------------VTISNEDFLYRGSHAGGSRFPTALAAHLNEYLTP 78

ACS3_1 --------------------------VTISNEDFLYRGSHAGGSRFPTALAAHLNEYLTP 78

ACS3_FungiDB DQLKEFADKS----------------VTISNEDFLYRGSHAGGSRFPTALAAHLNEYLTP 103

ACS3_FungiDB_1 DQLKEFADKS----------------VTISNEDFLYRGSHAGGSRFPTALAAHLNEYLTP 103

ACS2 KELIQHMTQNVKPPSHQYWVHANGFKLTITSHSLTCGDGFSGSHRLRDVLARFINRNFNP 105

ACS2_1 KELIQHMTQNVKPPSHQYWVHANGFKLTITSHSLTCGDGFSGSHRLRDVLARFINRNFNP 105

P.citrinum DELLEFINKK----------------LELPAKYLTYNDGGGGSSRLKAAIAAFLNHNLKP 95

ACS1 DVLRKHIHDN----------------LALTNPAFTYGDGTTGTKQVKKSVSRFLTKHLKP 92

ACS1_1 DVLRKHIHDN----------------LALTNPAFTYGDGTTGTKQVKKSVSRFLTKHLKP 92

: . * .. :: .: .

T.aestivum KARFDPDRVVMSGGATGAQETLAFCLANPGEAFLVPTPYYPGFDRDCCWRSGVKLLPIEC 159

ACS3 SSPITPDMIRCVGAATAMHDILAWGVADPGDGVLTSRPVYGRFELDFGNKSQAKVVYSDN 138

ACS3_1 SSPITPDMIRCVGAATAMHDILAWGVADPGDGVLTSRPVYGRFELDFGNKSQAKVVYSDN 138

ACS3_FungiDB SSPITPDMIRCVGAATAMHDILAWGVADPGDGVLTSRPVYGRFELDFGNKSQAKVVYSDN 163

ACS3_FungiDB_1 SSPITPDMIRCVGAATAMHDILAWGVADPGDGVLTSRPVYGRFELDFGNKSQAKVVYSDN 163

ACS2 HEAVTNDQLIITSGVGRAIELSGFSLCDKGDGVLLGRPHYGNFPIDFGYRAEAKIIGVSF 165

ACS2_1 HEAVTNDQLIITSGVGRAIELSGFSLCDKGDGVLLGRPHYGNFPIDFGYRAEAKIIGVSF 165

P.citrinum VIPLEPSHIMATNGVSSAIEHVAWSFADPGEGILLGRPYYGMFIPDMSLRTGSSVVPVSF 155

ACS1 FKAIEPAHITMTNGCSAAIEHLSWAVANPGDGILLGQPYYGTFVPDLTARFGAKLLPVAF 152

ACS1_1 FKAIEPAHITMTNGCSAAIEHLSWAVANPGDGILLGQPYYGTFVPDLTARFGAKLLPVAF 152

. : .. : .: ..: *:..* * * * * : .::

T.aestivum HSSNDFRITREA--VVAAYEGARSSGVRVKGILITNPSNPLGTTADRATLAMLATFATEH 217

ACS3 KTEEAFQDGIIDHFEEALLRSS-EAGIHVKMVLIVNPHNPLGRCYPKSTLVKIMQFCQKH 197

ACS3_1 KTEEAFQDGIIDHFEEALLRSS-EAGIHVKMVLIVNPHNPLGRCYPKSTLVKIMQFCQKH 197

ACS3_FungiDB KTEEAFQDGIIDHFEEALLRSS-EAGIHVKMVLIVNPHNPLGRCYPKSTLVKIMQFCQKH 222

ACS3_FungiDB_1 KTEEAFQDGIIDHFEEALLRSS-EAGIHVKMVLIVNPHNPLGRCYPKSTLVKIMQFCQKH 222

ACS2 GDVDPFSIEAVELYEKTLTDAQ-DQGIRVKALLLCNPHNPLGRCYTPEVLQAYMRLCQKH 224

ACS2_1 GDVDPFSIEAVELYEKTLTDAQ-DQGIRVKALLLCNPHNPLGRCYTPEVLQAYMRLCQKH 224

P.citrinum GELDPLSVEGVDKYEEALLEFHRTTGKKVKALMLAHPHNPLGRCYSREVLVKLMRLCQKY 215

ACS1 GEVDPLGEAAVAEYEKVILEAQ-AQGTRVSGLVISHPHNPLGRCYSRSVLIAFMKLCQKY 211

ACS1_1 GEVDPLGEAAVAEYEKVILEAQ-ARGTRVSGLVISHPHNPLGRCYSRSVLIAFVKLCQKY 211

: : . * :*. ::: :* **** .* :. ::

T.aestivum RVHLICDEIYAGSVFAKP-----EYVSIAEVIEHDAP-GADRDLIHIAYSLSKDFGLPGF 271

ACS3 RLHLLSDEIYACSVFNSD----EPATPFTSILSIDSTNLIDPDLLHVTYGLSKDFGAAGL 253

ACS3_1 GLHLLSDEIYACSVFNSD----EPATPFTSILSIDSTNLIDPDLLHVTYGLSKDFGAAGL 253

ACS3_FungiDB RLHLLSDEIYACSVFNSD----EPATPFTSILSIDSTNLIDPDLLHVTYGLSKDFGAAGL 278

ACS3_FungiDB_1 GLHLLSDEIYACSVFNSD----EPATPFTSILSIDSTNLIDPDLLHVTYGLSKDFGAAGL 278

ACS2 SLHLLSDEIYALSVWEND--NVPEAPGFTSVLSIDTNGLIDVNLVHALWGMSKDFGANGI 282

ACS2_1 SLHLLSDEIYALSVWEND--NVPEAPGFTSVLSIDTNGLIDVNLVHALWGMSKDFGANGI 282

P.citrinum QVHFISDEIYALSVFENTVDEHPPPVKFESALSIDLTGIIDPRLVHVLWGMSKDFGANGI 275

ACS1 EMHFISDEIYALSVWTNTVDQHPLSVPFESALSIDTTDIIDTDRVHVLWGMSKDFGANGI 271

ACS1_1 EMHFISDEIYALSVWTNTVDQHPLSVPFESALSIDTTDIIDTDRVHVLWGMSKDFGANGI 271

:*::.***** **: . : . :. * * :* :.:***** *:

T.aestivum RVGIVYSYNDAVVA-CARKMSSFGLVSSQTQLFLAKMLGDEEFMSRFLRESARRLAARHE 330

ACS3 RLGAIITRSQPVLRAI-EAAMRFHNPSGASLAIGNAMLEDRVWCRSFVDSSRSKLSQAHR 312

ACS3_1 RLGAIITRSQPVLRAI-EAAMRFHNPSGASLAIGNAMLEDRVWCRSFVDSSRSKLSQAHR 312

ACS3_FungiDB RLGAIITRSQPVLRAI-EAAMRFHNPSGASLAIGNAMLEDRVWCRSFVDSSRSKLSQAHR 337

ACS3_FungiDB_1 RLGAIITRSQPVLRAI-EAAMRFHNPSGASLAIGNAMLEDRVWCRSFVDSSRSKLSQAHR 337

ACS2 RIGCLITRNEAFMRAC-VANSDLSGPSSLSDLAAASILSDDAFLESFIKTNRLRLAENYK 341

ACS2_1 RIGCLITRNEAFMRAC-VANSDLSGPSSLSDLAAASILSDDAFLESFIKTNRLRLAENYK 341

P.citrinum RLGVIISQANRDIHMALTGPSLYSYASGITDHLTALILEDFDFTTRYIQQNQKLLSESYA 335

ACS1 RVGTIVSQANMSLHASIVAVGLYSSVSSISDHVTVNILEDDAFVESYIVENQKRLSAQYT 331

ACS1_1 RVGTIVSQANMSLHASIVAVGLYSSVSSISDHVTVNILEDDAFVESYIVENQKRLSAQYT 331

*:* : : : : *. : :* * : :: . *: :

T.aestivum LFTSGLREVGIGCL-GGNAGLFSWMDLRGMLREKTAE---------A----ELELWRVII 376

ACS3 HVTSQLKAMNIKYLPGSNAGFFVWIDLSPYLPSELDGEL-----------NQEFALAKRL 361

ACS3_1 HVTSQLKAMNIKYLPGSNAGFFVWIDLSPYLPSELDGEL-----------NREFALAKRL 361

ACS3_FungiDB HVTSQLKAMNIKYLPGSNAGFFVWIDLSPYLPSELDGEL-----------NQEFALAKRL 386

ACS3_FungiDB_1 HVTSQLKAMNIKYLPGSNAGFFVWIDLSPYLPSELDGEL-----------NREFALAKRL 386

ACS2 IVTEFLTNHDIPYKEGSNAGLFVWADLFAPNSALINDSVMKQDDAGAALMTMEENMTEIL 401

ACS2_1 MVTEFLTNHDIPYKEGSNAGLFVWADLFAPNSALINDSVMKQDDAGAALMTMEENMTEIL 401

P.citrinum YTANYLKEHGIEYATGCNAAFFVWMNLGKKYRELHP---------EDECENVGEQIMQRL 386

ACS1 RVVSWARKNQIEYAPGVNAAFFLWIDLGKYYMARHP---------GLETDDITDLIMSKL 382

ACS1_1 RVVSWARKNQIEYAPGVNAAFFLWIDLGKYYMARHP---------GLETDDITDLIVSKL 382

.. * * **.:* * :* :

T.aestivum RKVKLNVSPGTSFHCGEPGWFRVCHANMDDETMGVALSRIRDFVRQHQQQKAKAQRWAAR 436

ACS3 REAGVFLHPR-EEHSLEPGWFRIVYTQDP-RTVTEGLQRYVPSSFAVE-------TLK-- 410

ACS3_1 REAGVFLHPR-EEHSLEPGWFRIVYTQDP-RTVTEGLQRYVPSSFAVE-------TLK-- 410

ACS3_FungiDB REAGVFLHPR-EEHSLEPGWFRIVYTQDP-RTVTEGLQSSQGQNGNIV-------GWTLE 437

ACS3_FungiDB_1 REAGVFLHPR-EEHSLEPGWFRIVYTQDP-RTVTEGLQSSQGQNGNIV-------GWTLE 437

ACS2 LKERIFVASGGDFGTDVSGWYRIVFAHER-TYLLEGLERIVRAVKKFC-------RDS-- 451

ACS2_1 LKERIFVASGGDFGTDVSGWYRIVFAHER-TYLLEGLERIVRAVKKFC-------RDS-- 451

P.citrinum LQKKVFLASGFLFGSEKDGWFRIVFTQGH-DYLSVALERINAALEE-------------- 431

ACS1 MAKKIFLASGKGFGSEKPGWFRIVFSHDD-AYLDLGLERVINALL--------------- 426

ACS1_1 MAKKIFLASGKGFGSEKPGWFRIVFSHDD-AYLDLGLERVINALL--------------- 426

: : **:*: .:: : .*.

T.aestivum SHL-HLSLQRHGPMASQYHALSSPMAALLSPQSPLVHAAS 475

ACS3 ---------------------------------------- 410

ACS3_1 ---------------------------------------- 410

ACS3_FungiDB KRSTILAVNKVKEREGFYE--------------------- 456

ACS3_FungiDB_1 KRSTILAVNKVKEREGFYE--------------------- 456

ACS2 -KD-------------Y----------------------- 454

ACS2_1 -KD-------------Y----------------------- 454

P.citrinum ---------------------------------------- 431

ACS1 ---------------------------------------- 426

ACS1_1 ---------------------------------------- 426


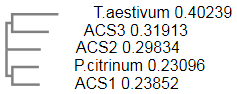


Supplementary Figure 3

Supplementary Figure 3: Clustal omega alignment of two functional ACC synthases from *Penicillium citrinum* (GenBank: BAA92149.1), *Triticum aestivum* (GenBank: AAB18416.1) and three ACC synthase candidate genes of *Fusarium graminearum* (ACS1 (XP_011323690.1), *ACS2* (XP_011327389.1), *ACS3* (XP_011328278.1), *ACS3* as annotated in Fungi DB (FGRAMPH1_01T27057)). The green highlighted amino acids are important for ACS activity. Blue highlighted amino acids indicate RNA modification during sexual development. For *ACS1* the probabilities of the three amino acid changes are 20% Q to R), 10% (M to V) and 17% (M to V) at position 379. In *ACS2* the probability of I to M change is estimated with 39% while the changes in *ACS3* have a lower likelihood with 4% (R to G) and 4% (Q to R).

ACD1 --MVTLPSPFSDIDRVQLLFNRPTDIEPLSRLTESVNNNVKLWIAREDRNSGLAFAGNKV 58

Trichoderma MATLNIPEPLASIPFESLLFG-PSPIQHLPRISAALGGKVTVYAKRDDCNSGFAYGGNKV 59

ACD2 MTVVTLPEPFASIPRENFLFG-ASPLQPLPRISAALGGKVNVYAKREDCNSGLAYGGNKV 59

:.:*.*::.* .:**. : :: * *:: ::..:*.:: *:* ***:*:.****

ACD1 RKLEYVLADALAQGADTVVTTGGIQSNHMCQTSAAAARLGLKVALYPADRVASNDAEYKY 118

Trichoderma RKLEYLAAEALSQGCDTLVSIGGVQSNHTRAVTAVAAKLGLKAATVQEHWVDWDDAGYEK 119

ACD2 RKLEYLAAEAQAEGCDTLVSIGGVQSNHTRAVTAVASKLGLKAATVQEHWVDWEDPGYEK 119

*****: *:* ::*.**:*: **:**** .:*.*::****.* . * :* *:

ACD1 LGNIQANAILGAETF------PVD--TAEETVITTLKDRGQKPYSIPPGASSHPLGGLGY 170

Trichoderma VGNIQLSRLMGGDVRLDPSLFGIEHKPTLANLKAELEGSGRKPYYIPAGASDHPLGGLGF 179

ACD2 VGNIQLSRLMGGDVRLDPSTFGIEHKTTLAKLKDELKSNGQKPYYIPAGASDHPLGGLGF 179

:**** . ::*.:. :: : .: *:. *:*** ** ***.*******:

ACD1 ARWAFELLEQEKKIGVTFDTIALVAGSCSTLGGLLAGLKLAQKEQIPGSKKRLIGFSVLH 230

Trichoderma ARWALEVEAQEKEMGVFFDTVIVCAVTGSTMAGMIAGFKLAQLKLG-SPKRKIIGIDASG 238

ACD2 ARWAFEVEAQEKELGIFFDTVIVCAVTGSTFAGMIAGFKLAQKKNG-SPARKIIGIDASG 238

****:*: ***::*: ***: : * : **:.*::**:**** : . :::**:..

ACD1 KSKKDVEALVLKTSRTTASKIGISPNEITADDFEINTSYIGDGYGQLNDSTAEAMKKLAR 290

Trichoderma KPKETF-DQVLRIAKFTAAKIGLSEADITEADVILDERFNAKIYGIPDETTIEAMKFGAR 297

ACD2 KVQQTF-DQVLRIAKNTAAKIGLSEDDITADDVILDPNYNAKVYGIPDETTLEAMRFGAA 297

* :: . **: :: **:***:* :** *. :: : .. ** :::* ***: *

ACD1 KEGILTDPVYTGKAFTGLLDLAKTGYLNGKNVLFLHTGGQAVLSAYPGLRE 341

Trichoderma TEAFITDPVYEGKSLAGMMGLIRNGEIAGGNVLYAHLGGQLALNAYSSLLD 348

ACD2 TEAFITDPVYEGKSLAGMMDLIKTGKIAGGNVLYAHLGGQLALNAYSSI-- 346

.*.::***** **:::*::.* :.* : * ***: * *** .*.** .:


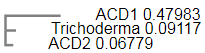


Supplementary Figure 4: Clustal omega alignment and a phylogenetic tree of a confirmed ACC deaminase of *Trichoderma asperellum* (ACX94231.1) and both ACC deaminase candidate genes of *Fusarium graminearum* (FGSG_02678 = *ACD1* and FGSG_12669 = *ACD2*).

M ACS1 ACS3 ACD1 ACD2 C ACS2


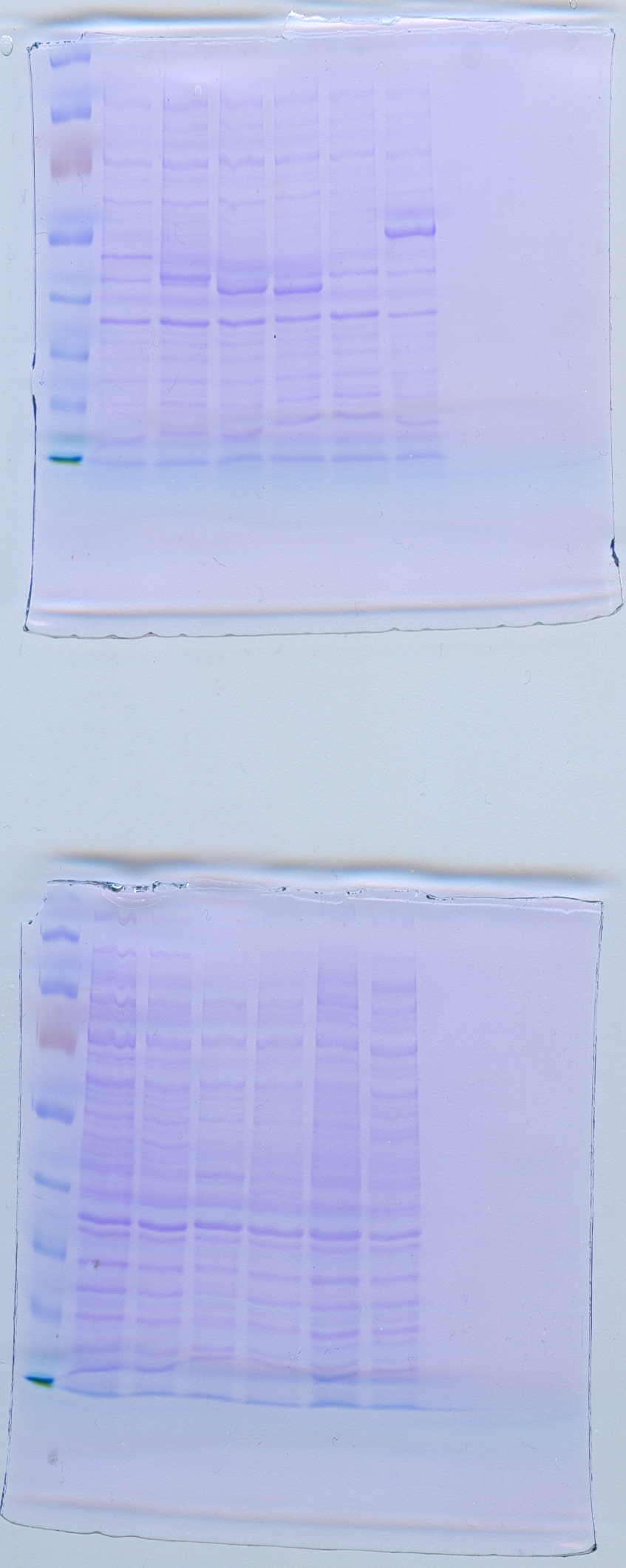


130 kDa

100 kDa

70 kDa

55 kDa

40 kDa

Supplementary Figure 5: Confirmation of expression of *ACS* and *ACD* candidate genes of *F. graminearum* in *E. coli*. C, vector control. M, marker


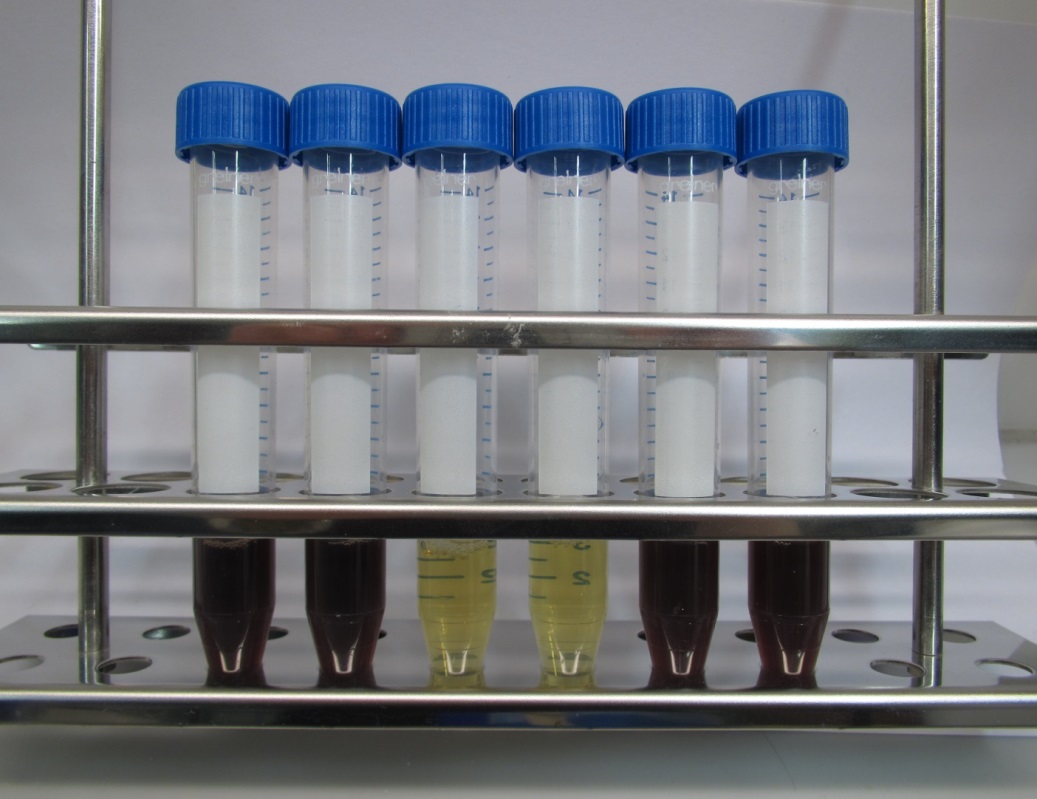


ACD2

*P. putida*

Negative

2

1

2

1

1

2

Supplementary Figure 6: Colorimetric assay of Acd2 activity of *F. graminearum*, a positive control of *Pseudomonas putida* and a negative control;


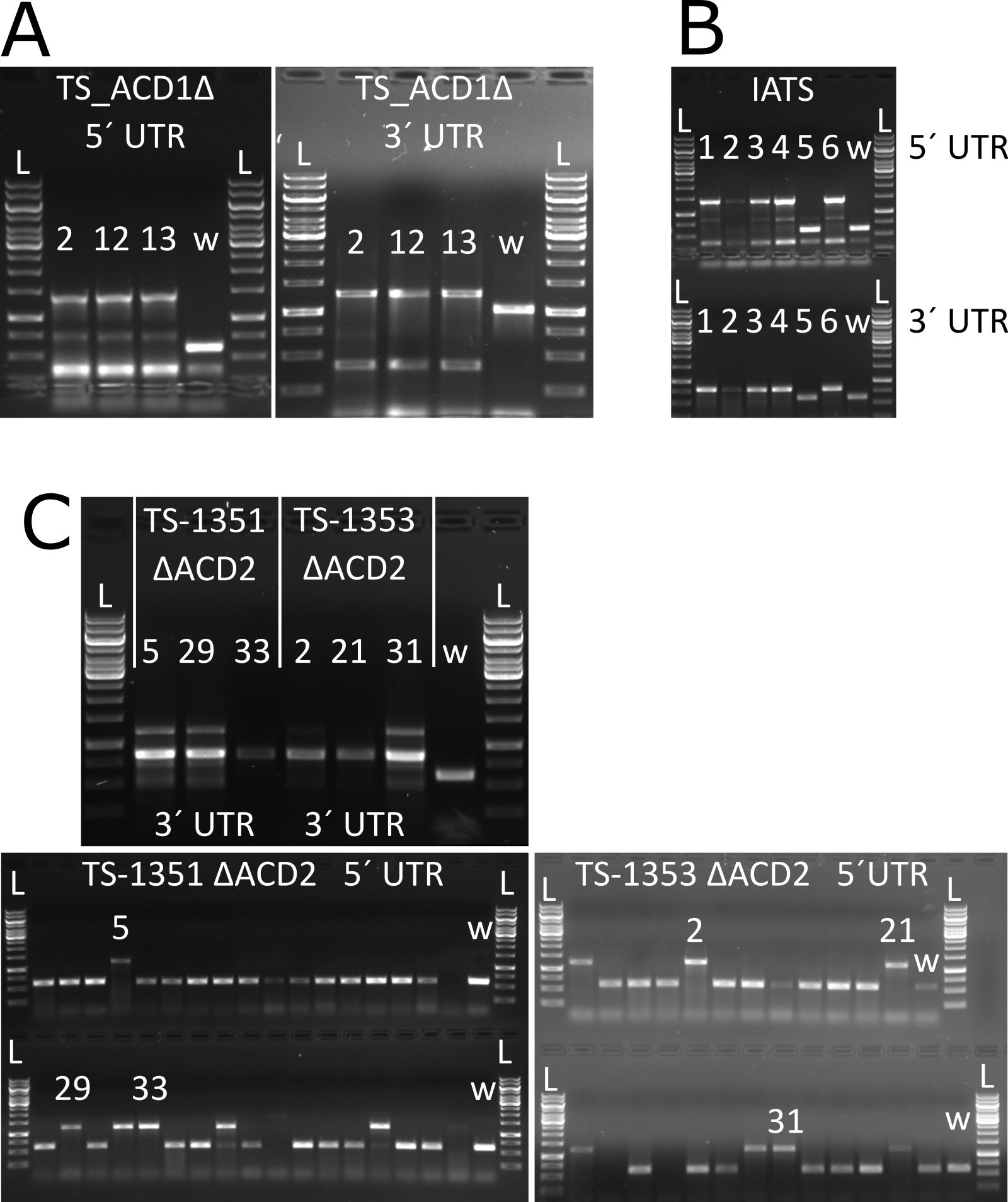


Supplementary Figure 7: Knock out screening using multiplex PCR; A) ∆ACD1 mutant: the expected lengths for the 5´ screening are 620 bp and 1434 bp for the wild type and the knock out strain, respectively (left side). Expected lengths are 1033 bp and 1287 bp for the 3´screening (right side); B) ∆ACD2 mutant: expected lengths for the 5´ screening (top) are 614 bp (w) and 1403 bp (∆) for the wild type and the knock out strain, respectively. For the 3´screening (bottom) 608 bp (w) and 804 bp (∆); C) ∆∆ACD1,2 mutant: expected lengths for the 3´-screening (top image) of the three double knock-out strains (derived from independent single knock out strains) used in this study is shown (w = 608; ∆ = 833 bp); expected lengths for the 5´screening of the ACD2 knock-out in two independent confirmed ∆ACD1 knock out strains (w = 614 bp; ∆ = 1245 bp) (bottom left and right images); w = wild type; L = Gene Ruler 1 kb DNA ladder (Thermo Scientific).

(A)

(B)

Supplementary Figure 8: A) Mean values of DON and D3G toxins including standard deviation extracted from wheat ears infected with PH-1, Δacd1 and Δacd2 knock- out strains. B) Mean values of toxins including standard deviation extracted from ears inoculated with PH-1 or six independently derived double knock out strains from two independent Δacd1 strains
